# Integrative Taxonomy Approach Reveals Cryptic Diversity within the Phoretic Pseudoscorpion Genus *Lamprochernes* (Pseudoscorpiones: Chernetidae) †

**DOI:** 10.3390/insects14020122

**Published:** 2023-01-25

**Authors:** Jana Christophoryová, Katarína Krajčovičová, František Šťáhlavský, Stanislav Španiel, Vera Opatova

**Affiliations:** 1 Department of Zoology, Faculty of Natural Sciences, Comenius University, Mlynská Dolina, Ilkovičova 6, 842 15 Bratislava, Slovakia; 2 Department of Zoology, Faculty of Science, Charles University, Viničná 7, 128 44 Praha, Czech Republic; 3 Institute of Botany, Slovak Academy of Sciences, Dúbravská Cesta 9, 845 23 Bratislava, Slovakia

**Keywords:** cytogenetics, DNA barcoding, morphological stasis, morphometrics, phoresy, species delimitation

## Abstract

**Simple Summary:**

Pseudoscorpions are a wide-spread, but often-overlooked group of animals. They are generally of small body size and rather homogeneous appearance. The genus *Lamprochernes* is well-defined, but delineating species within the genus can be quite challenging. It comprises several morphologically similar species with wide and overlapping distributions, commonly found under tree bark and in anthropogenic habitats such as composts and manure heaps. In this paper, we implemented an integrative approach combining molecular, cytogenetic and morphological analyses in order to assess species boundaries in European *Lamprochernes* populations. Our results uncover an existence of a new *Lamprochernes* species—*Lamprochernes abditus* sp. nov., which can be distinguished from its closest relative only by molecular and cytogenetic differences, or alternatively, by a complex multivariate morphometric analysis involving other *Lamprochernes* species. Our results also suggest that the genus *Lamprochernes* likely uses phoresy (a non-permanent interaction in which one organism (phoront) attaches itself to another (host) for the purpose of travel) very efficiently for its dispersal.

**Abstract:**

Pseudoscorpions represent an ancient, but homogeneous group of arachnids. The genus *Lamprochernes* comprises several morphologically similar species with wide and overlapping distributions. We implemented an integrative approach combining molecular barcoding (*cox1*), with cytogenetic and morphological analyses in order to assess species boundaries in European *Lamprochernes* populations. The results suggest ancient origins of *Lamprochernes* species accompanied by morphological stasis within the genus. Our integrative approach delimited three nominal *Lamprochernes* species and one cryptic lineage *Lamprochernes abditus* sp. nov. Despite its Oligocene origin, *L. abditus* sp. nov. can be distinguished from its closest relative only by molecular and cytogenetic differences, or alternatively, by a complex multivariate morphometric analysis involving other *Lamprochernes* species. The population structure and common haplotype sharing across geographically distant populations in most *Lamprochernes* species suggest that a phoretic manner of dispersal is efficient in this group.

## 1. Introduction

The speciation process is not always accompanied by morphological changes. However, morphologically cryptic species can be often distinguished e.g., by their genetic, ecological and behavioral differentiation [1]. Although additional resources may be required for identifying these species, not accounting for the cryptic diversity could have serious consequences for human health [2,3], agriculture [4], conservation management [5,6,7] and our understanding of evolution [8]. Despite these potentially broad impacts, what constitutes a cryptic species and whether it represents only a temporary state is still a matter of debate [9,10]. Morphological differences are often found among cryptic species after e.g., genetic data-guided specimen clustering. Phenotypic homogeneity may thus be predominantly related to character-interpretation issues [11,12]. Species crypsis can be a consequence of unrelated processes, such as morphological stasis, convergence and parallelism [13]. It can therefore involve both recently evolved and ancient taxa. The use of genetic data is thus essential both from the perspective of an alpha taxonomy [1], i.e., detecting and delimiting cryptic lineages, as well as for understanding the underlying evolutionary processes of cryptic speciation [9,10].

Barcoding has been established as an accessible and cost-effective method that can easily provide insights into the diversity of particular groups [14], habitats [15,16,17], or large geographic regions [18,19,20]. The European biota has been extensively studied, and as a result, its diversity and evolutionary history are among the best understood world-wide. This is particularly true for western, central and northern parts of Europe [21,22,23,24]; however, ongoing barcoding efforts continue to elucidate the existence of overlooked arthropod diversity even in these well-studied regions [25,26,27]. Arachnids are no exception [28,29], due to a combination of their often challenging morphology-based species recognition and taxonomic neglect [30].

Slight morphological differences can be mistaken for intraspecific variation among sibling species with overlapping distributions, and cryptic species thus cannot be detected unless other lines of evidence are also considered [31]. Molecular approaches are invaluable in the species discovery step [32], but caution should be taken into account during the interpretation of the results. For instance, genetic structure can be mistaken for species boundaries and lead to taxa over-splitting [33]. Subsequent validation of putative lineages by other sources of evidence is thus essential [32], which results in a significant number of cryptic species remaining formally undescribed.

Pseudoscorpions represent the fourth most diverse order of the class Arachnida, comprising over 4000 described species [34]. They occur in most terrestrial habitats [35], but due to their generally small body size (~2–4 mm) [36], pseudoscorpions are often overlooked. Although their deep-level phylogenetic relationships are now better understood [37], our knowledge of pseudoscorpion intra-familial relationships and phylogeography is still fairly limited [38,39,40,41,42,43,44,45]. Pseudoscorpions often show a relatively uniform external morphology among closely related species [46,47], but they can exhibit a great plasticity in terms of their karyotypes [48,49,50,51,52,53]. Their interspecific karyotype variability is high; a diploid chromosome number varies from 2n = 7 to 143 [50,51]. Therefore, cytogenetic data can be used to resolve taxonomic problems and have the potential to reveal cryptic species [54].

The genus *Lamprochernes* Tömösváry, 1883 belongs to the cosmopolitan and species-rich family Chernetidae. Alongside seven other genera, it composes the morphologically well-defined subfamily Lamprochernetinae [55]. *Lamprochernes* comprises nine species, of which five occur in Europe [34]. *Lamprochernes leptaleus* (Navás, 1918) has only been recorded in Spain and *L*. *moreoticus* (Beier, 1929) in Greece, while *L*. *chyzeri* (Tömösváry, 1883) and *L. nodosus* (Schrank, 1803) are distributed across the entirety of Europe. *Lamprochernes savignyi* (Simon, 1881) is known from Spain, the United Kingdom, Ireland, Denmark, Switzerland and Germany [29,34,56]. The last three species also occur outside of Europe [34], likely owing their distribution to a combination of phoretic dispersal (i.e., hitchhiking on a vagile carrier) and human introductions. They can be found under tree bark and in bird nests, but they are also common in anthropogenic habitats such as composts and manure heaps [57,58,59,60,61,62]. Such habitats may contain thousands of individuals and serve as sources for mass propagation [63].

Although the genus *Lamprochernes* is both well-defined morphologically [62,64] and well supported at the molecular level [29], delineating species within the genus can be quite challenging. In several cases, the original descriptions of European *Lamprochernes* lack details [65,66,67]. Additionally, the taxonomy may be further complicated by poor character definition. For instance, *L. moreoticus* was described based on two adults and its diagnostic character was a larger body size compared to the remaining species, whereas its distinguishing trichobothria pattern was added later [64,67,68]. Even more complicated is the *L*. *leptaleus* case. Its description is rudimentary, based on a single individual and does not provide any evidence for the species’ placement within the genus *Lamprochernes* [65]. The only wide-spread species that has undergone a thorough revision and redescription is *L. savignyi* [62]. Distinguishing the remaining commonly occurring species, *L*. *chyzeri* and *L*. *nodosus,* is also challenging. Both species occur in similar habitats and their identification is based on a few overlapping characters [36,63], such as the length of the palpal femur and the shape of palpal trochanter protuberance, which may be rather subjective to interpretation [69]. Clear distinction, i.e., non-overlapping palpal femur lengths, was assumed by Mahnert [70], but this observation is in direct disagreement with subsequent assessment of the character’s variability [69]. It is thus clear that both species deserve a thorough taxonomic reconsideration.

## 2. Material and Methods

### 2.1. Taxonomic Sampling and Species Delimitation Workflow

**Figure 1:**
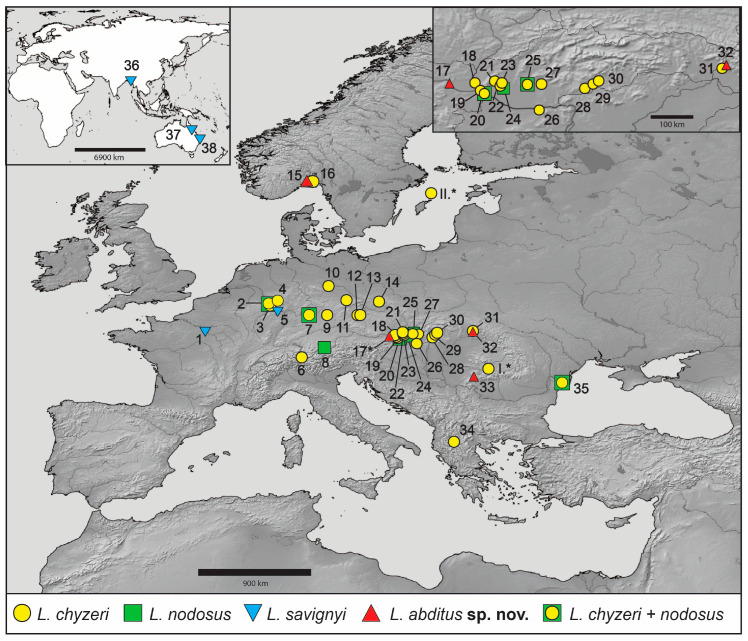
Map showing sampling locations of *Lamprochernes* species, including data from public databases and museum collections, analyzed in this paper. Different symbols correspond to different *Lamprochernes* species depicted at the **bottom**. Sampling locations of type material are marked with asterisks; specimens marked with roman letters were not analyzed molecularly (I. *L*. *chyzeri*, II. *L*. *mjobergi*). **Upper left** corner insert shows the sampling locations in Asia and Australia; the **upper right** insert shows sampling locations in Austria, Slovakia and Ukraine in detail. All maps were created with the help of an online version of SimpleMappr.

*Lamprochernes* individuals were collected by compost sifting and extraction and from on or under tree bark across Europe (Table S1, Figure 1). The specimens were preliminarily determined, following the literature [56]. Distribution maps were created in SimpleMappr [71] and edited with Adobe Illustrator. Geographic distances among sample localities were obtained in Geographic Distance Matrix Generator [72]. To delimit *Lamprochernes* species, we first implemented a molecular data-based species discovery step, which was followed by a species validation step combining multispecies coalescent analyses (molecular data), morphometric analyses (morphology data) and cytogenetic analyses (karyotype data).

### 2.2. Molecular Protocols and Phylogenetic Analyses

Whole genomic DNA was extracted from the samples using the DNeasy Tissue Kit (Quiagen, Hilden, Germany) following the manufacturer’s protocol. Partial fragment of mitochondrial gene Cytochrome oxidase I (*cox1*) (the animal barcode) was amplified for all the samples with the primer combination C1-J-1490/C1-N-2198 [73]. The PCR products were purified using MinElute PCR Purification Kit (Quiagen) and sequenced in both directions by Macrogen Inc. (Seoul, Republic of Korea). The chromatograms were assembled, edited and aligned in Geneious v. 5.6. [74].

Phylogenetic trees for the tree-based species delimitation methods were inferred from a dataset combining our barcode data with additional *Lamprochernes* sequences from GenBank and BOLD databases (accession numbers are provided in Table S1). Because the monophyly of European representatives of *Lamprochernes* has never been disputed (but see Introduction for information about *L. leptaleus* (not sampled in this study)) and the genus received a high bootstrap support (=91) in barcoding analyses involving extensive taxon sampling [29], we opted for a smaller selection of outgroup taxa to root our trees. We used closely related *Chernes hahnii* (C.L. Koch, 1839) (Chernetidae, subfamily Chernetinae), *Chelifer cancroides* (Linnaeus, 1758) (family Cheliferidae) and the distantly related *Neobisium polonicum* Rafalski, 1936 (family Neobisiidae). To ensure the correct alignment of all *Lamprochernes* terminals with the outgroup taxa sequences, the online version (http://translatorx.co.uk/ (accessed on 1 February 2022)) of Translator X [75], a program which considers amino acid information of protein-coding genes, was used to realign the preliminary data matrix obtained in Geneious. The resulting alignment (“Lampro_all” data matrix) was visually checked to confirm the absence of poorly aligned positions and stop codons. The matrix was further reduced to contain only unique haplotypes (“Lampro_reduced” data matrix).

The best partitioning scheme and evolutionary models for each *cox1* codon position of the “Lampro_reduced” data matrix (45 unique *Lamprochernes* haplotypes, 3 outgroup taxa) were selected using the greedy algorithm in Partition Finder 2 [76]. Maximum likelihood (ML) analyses were conducted in RaxML v 8.2.9 [77]. An independent GTR + G substitution model was assigned to each codon position. The best ML tree was selected from 1000 independent searches. Bootstrap support of the nodes was assessed from 1000 replicates. The Bayesian inference (BI) analyses were conducted in BEAST v. 2.5. [78]. An independent evolutionary model was defined for each codon position as follows: F81 + I to 1st, HKY + G to 2nd and TRN + G to 3rd *cox1* position. Two independent runs of 5 × 10^7^ generations, with parameter and tree resampling every 1000 generations, were used to infer *Lamprochernes* intrageneric relationships under the Yule model as tree prior. Convergence and chain mixing was assessed by the standard deviation of split frequencies (<0.01) and ESS values were summarized in TRACER v. 1.5 [79]. The first 20% of the generations were discarded as a *burn-in* in BEAST accompanying program LogCOMBINER; tree topology was annotated in TREEANNOTATOR. The trees were visualized and manipulated with the program FigTree v. 1.3.3 [80].

### 2.3. Molecular Species Delimitation

We implemented both species-discovery and species-validation approaches in our molecular species delimitation. First, we employed the statistical parsimony (SP) haplotype network analysis [81] implemented in TCS v 1.21 [82] to delimit species (95% parsimony criterion) and infer their intraspecific structure.

Second, we used three tree-based delimitation methods. We implemented both the Poisson Tree Process (PTP) as well as its Bayesian implementation (bPTP) [83]. The analyses were run for 5 × 10^5^ generations on the bPTP webserver (https://species.h-its.org/ptp/ (accessed on 17 March 2022)), using the topology recovered in RAxML analyses. Outgroup taxa (*N. polonicum, C. cancroides* and *C. hahnii*) were removed prior to the analyses to improve the delimitation results [83]. Generalized mixed Yule coalescent (GMYC) [84] analysis with a single threshold was carried out in the R environment (http://www.r-project.org (accessed on 17 March 2022)) using the SPLITS package [85]. The input tree was obtained in BEAST from two independent runs of 3 × 10^7^ generations. The analyses were performed on “Lampro_reduced” data matrix without outgroups (45 *Lamprochernes* terminals) under the Coalescent–constant size model as a tree prior. The convergence was assessed in TRACER; the first 20% of the generations were discarded as a *burn-in*.

As a third and final discovery step approach, we implemented Assemble Species by Automatic Partitioning (ASAP) [86], a novel distance method based in hierarchical clustering. The analyses were performed remotely on the ASAP delimitation web server (https://bioinfo.mnhn.fr/abi/public/asap/ (accessed on 17 March 2022)) under both Kimura (K80) substitution model [87] and simple p-distances.

The molecular-based component of the species validation step was carried out in the BPP v 4.1.4 [88] under the multi species coalescent model (MSC). We conducted species-delimitation model inference on a *Lampro_all* data matrix without outgroups (155 *Lamprochernes* terminals) and fixed guide tree (A10 inference in BPP) [89,90]. The guide tree ((*L. nodosus*, *L. savignyi*), (*L. chyzeri*, *L. abditus* sp. nov.)) reflected *Lamprochernes* topology recovered in the previous ML and BI analyses. The individuals were assigned into species based on the congruent results of the discovery step combined with the results of the cytogenetic and multivariate morphometric analyses (see results). All delimitation models were given equal probabilities. We assigned inverse-gamma prior θ ~ IG (3, 0.017) for all θ parameters and τ ~ IG (3, 0.1) for the root age. The IG $\left(\alpha ,\;\beta \right)$ values for β were calculated from the data [88], while α=3 represented a diffuse prior. The first 100,000 generations were discarded as a *burn-in*, 100,000 samples were collected from the MCMC run, sampling every 10th generation. Three independent runs were conducted to assess the species delimitation outcome and convergence of the analyses.

Genetic distances among and within the delimited *Lamprochernes* species were calculated via both the uncorrelated p-distance and the Tamura-Nei distance model [91] in Mega 11 [92].

### 2.4. Divergence Time Analyses

Divergence times among the delimited species were estimated in BEAST. The analyses were run on a newly created matrix (“Lampro_time”), comprising four *Lamprochernes* species and six outgroups. The outgroups (*C. hahnii*, *Withius* sp., *Oratemnus curtus* [39], *C. cancroides*, *Cheiridium museorum* [29] and *N. polonicum*) were selected to match the lineages represented in the divergence time analyses of Benavides and collaborators [37]. Due to the accelerated mutation rate in pseudoscorpions, the general arthropod mitochondrial substitution rate of 2.3% [93] was not implemented in our analyses; instead, we calibrated our analyses by a combination of fossil record and ages inferred by Benavides and collaborators [37]. The age of *Heurtaultia rossiorum* (100 Million years ago (Ma)) [94] was assigned as a minimum age of the split between Cheliferidae and Withiidae + Atemnidae clade (*C. cancroides* − *Withius* sp. + *O. curtus*). The prior was assigned a log normal distribution with offset = 100 (mean in real space), M = 10, S = 1.25 to include the confidence interval of Cheliferidae − Withiidae + Atemnidae split (log normal clock) inferred by Benavides and collaborators [37] in their transcriptomic analyses. Normal distribution priors, with ∑ = 10 to include the confidence intervals, were assigned to the ages of all the secondary calibration points [37] as follows: Withiidae − Atemnidae, mean = 93 Ma; Chernetidae, mean = 101 Ma; Cheliferoidea, mean = 128 Ma; Cheliferoidea + Cheiridiidae, mean = 128 Ma. The topology of outgroups was constrained to match the corresponding part of the chronogram inferred by Benavides and collaborators [37]. A GTR + G substitution model selected by Partition Finder and log normal relaxed clock were assigned to the *cox1* dataset, which was treated as a single partition. A Yule model was set as a tree prior. The analyses were run three times independently for 3 × 10^5^ generations with parameter sampling every 1000 generations.

### 2.5. Cytogenetic Analyses

We used chromosome slides obtained from several individuals of each putative species for the cytogenetic analyses (Table S1). The chromosome preparations were made from the gonads using the technique previously applied in chernetid pseudoscorpions [95]. During this process, the gonad was hypotonized in 0.075 M KCl for 15 min, fixed in methanol:glacial acetic acid (3:1) for 20 min and dissociated in a drop of 60% acetic acid. The suspension was spread on a microscope slide on a warm histological plate (45 °C). Finally, the chromosomes were stained in 5% Giemsa solution in Sörensen phosphate buffer for 30 min and documented through an Olympus AX70 Provis microscope using an Olympus DP72 camera and QuickPHOTO CAMERA v.2.3 software (Promicra, Prague, Czechia). For each species, five nuclei were measured using the plug-in Levan for ImageJ [96]. This plugin utilizes the chromosome classification according to Levan, et al. [97] and Green and Sessions [98]. We analyzed subtelocentric and telocentric chromosomal types together, due to difficulties in locating the exact position of centromere in the distal region of small chromosomes. We classified these types as one-armed chromosomes, whereas metacentric and submetacentric chromosomes were considered as bi-armed.

In our cytogenetic analyses, we also implemented fluorescent in situ hybridization (FISH) with an 18S rDNA probe for identification of rDNA clusters in all karyotyped individuals of *Lamprochernes abditus* sp. nov., *L. chyzeri* and *L. nodosus*. The probe was prepared from the scorpion *Euscorpius sicanus* (C. L. Koch, 1837) (Euscorpiidae), according to Šťáhlavský, et al. [99]. This probe was labelled with biotin-14-dUTP (Roche) using Nick Translation Kit (Abbott Molecular), according to the manufacturer’s protocol. FISH protocol followed Sadílek, et al. [100]. During the procedure, chromosome preparations were treated with RNase A and denatured at 68 °C for 3 min 30 s in 70% formamide in 2× SSC. Biotin-labelled probe was hybridized on the chromosomal preparation overnight and the signal was detected by streptavidine-Cy3. The chromosomes were counterstained by Fluoroshield™ with DAPI (Sigma-Aldrich, St. Louis, MO, USA) and photographed using an ORCA-AG monochromatic camera (Hamamatsu, Shizuoka, Japan) on an Olympus IX81 microscope. The images were pseudocolored (red for Cy3 and blue for DAPI) and superimposed with ImageJ software (https://imagej.nih.gov/ij/ (accessed on 5 May 2022)).

### 2.6. Morphological Analyses

Pseudoscorpions were studied as temporary slide mounts prepared by immersing the specimens in lactic acid for clearing. The pedipalps and legs I and IV were dissected for detailed study. Each specimen and its dissected body parts was preserved in 75% ethanol after examination. Ten specimens representing all morphologically analyzed putative species were compared by Scanning electron microscopy (SEM) at the Laboratory of Confocal and Fluorescence Microscope, Faculty of Science, Charles University (Table S1). SEM photographs were taken with a JEOL JSM-6380LV microscope. The remaining *Lamprochernes* specimens were photographed using a Leica DM1000 compound microscope with an ICC50 Camera Module (LAS EZ application, 1.8.0) for the purposes of morphological and morphometric analyses and species description. Measurements were taken from digital images using the AxioVision 40LE application. The measurements (see Tables S2 and S9) were taken using the reference points proposed by Chamberlin [101] and reported in millimeters (mm). The pedicel was included in the chela and chelal hand length measurements. All ratios are presented either as length/width ratios (carapace, chelicera and pedipalp) or as length/depth ratios (legs). Drawings were made using a Leica DM1000 drawing tube. Morphological terminology follows Chamberlin [101], with amendments proposed by Harvey [102] and Judson [103]. Nomenclature for all taxa follows WPC [34].

The specimens are deposited in the zoological collections of the Department of Zoology, Comenius University in Bratislava, Slovakia (KO/PK specimen number/35); the Department of Zoology, Charles University, Czech Republic (CZ_) and in the zoological collections of the Naturhistorisches Museum Wien, Austria (NHMW-Zoo-AR number) (for details see Table S1).

*Lamprochernes* type material was borrowed from the following institutions: Hungarian Natural History Museum (HNHM Pseudoscorp-392, *L. chyzeri*), Swedish Museum of Natural History (NHRS-TOBI000005234, *L. mjobergi* (Tullgren, 1909), junior synonym of *L*. *chyzeri*) (for details see Table S1).

### 2.7. Morphometric Analyses

Methods of multivariate morphometrics [104] were used to examine the morphological differences among 103 individuals delimited as three putative species in the species discovery step of molecular species delimitation analyses: *Lamprochernes abditus* sp. nov., *L. chyzeri* and *L. nodosus*. Additionally, 17 individuals were used in molecular analyses and for taxonomic descriptions, but were excluded from the morphometric dataset because of damaged body parts. The type material of *L*. *chyzeri* (6 individuals) and *L*. *mjobergi* (1 individual) could not be used in the molecular analysis, but were used for taxonomic descriptions.

The measured and scored morphological characters included those reported as taxonomically relevant within the genus in the identification keys and other treatments. The character shape of protuberance on palpal trochanter, the main character used for taxonomic identification of the studied samples, was omitted from the statistical analyses to avoid circular reasoning. Altogether, 108 quantitative characters were measured or scored, 51 were continuous (Tables S2 and S9) and 57 were discrete (see morphological descriptions in Results and “Supplementary Material_Description of *Lamprochernes chyzeri* and *nodosus*” and Table S9). Setae and lyrifissures on palpal and pedal coxae were added to the descriptions of all species, but were not statistically analyzed.

Only 56 characters (Table S7) were retained for further statistical analyses (14 characters were redundant (ratios) and 21 characters were invariable among measured specimens). All analyses were performed independently both on complete and reduced datasets of males and females. The complete dataset of males comprised 66 individuals in three groups (4, 58 and 4 of *L. abditus* sp. nov., *L. chyzeri*, and *L. nodosus*, respectively) and 56 characters. The complete dataset of females comprised 37 individuals in three groups (5, 21 and 11 of *L. abditus* sp. nov., *L. chyzeri*, and *L. nodosus*, respectively) and 56 characters. Reduced datasets contained only a selection of characters for both sexes, 21 characters for males and 9 for females, respectively. These characters were selected based on results of stepwise discriminant analyses (see below).

Pearson [105] and non-parametric Spearman correlation coefficients [106] were computed to reveal the correlation structures among the characters in all the datasets in order to screen for very high correlations (above 0.95) that could distort further multivariate analyses. Highly correlated characters and characters which were invariable in one or more groups/taxa were removed from further analyses. 

Discriminant analyses [104,107] were employed to assess the morphological differentiation among the three morphologically analyzed *Lamprochernes* lineages. The discriminant analyses included stepwise discriminant analysis (stepwise DA), canonical discriminant analysis (CDA) and linear classificatory discriminant analysis (LDA). Stepwise DA was used to remove redundant or unnecessary characters present in the complete datasets of both sexes and to identify the most useful characters. CDA 1 and LDA 1 (for males) and CDA 2 and LDA 2 (for females) were then carried out with the reduced datasets containing only a selection of characters contributing the most to the differentiation of the predefined groups (the three *Lamprochernes* species). CDA 1 and 2 were used to show the extent of morphological differentiation among the predefined groups and to identify the most important differentiating characters. The 95% confidence ellipses were drawn on CDA diagrams. The eclipses predict the regions around taxa, where a new independent observation from the respective taxon would be placed. The LDA 1 and 2 were used to derive linear classification functions and acquire a simple classification criterion that could be used for the taxonomic determination of an unknown individual (the classification into one of the three species).

The obtained linear classification functions were used to test the assignment of type specimens of *L*. *chyzeri* and *L*. *mjobergi* into one of the three *Lamprochernes* lineages.

All analyses were performed in R 4.0.0 software [108] using the MorphoTools2 package (https://github.com/MarekSlenker/MorphoTools2| (accessed on 5 May 2022)) [109].

## 3. Results

### 3.1. Molecular Protocols and Phylogenetic Analyses

A matrix of 158 terminals (“Lampro_all”), representing 155 *Lamprochernes* individuals and three outgroups (*N. polonicum*, *C. cancroides* and *C. hahnii*), was created for this study. *Cox1* sequence data (618 bp) were newly obtained for 132 individuals; the remaining sequences were obtained from public databases. Detailed information about the locality data of collected individuals alongside GenBank and BOLD accession numbers is provided in Table S1. After collapsing all terminals into unique haplotypes, a reduced matrix (“Lampro_reduced”) comprising 48 terminals (45 ingroup taxa, 3 outgroups), was created for downstream phylogenetic and species delimitation analyses (species discovery step). The matrix consisted of 618 bp, of which 217 represented parsimony informative positions (pi); 174 pi sites were within ingroup taxa.

**Figure 2:**
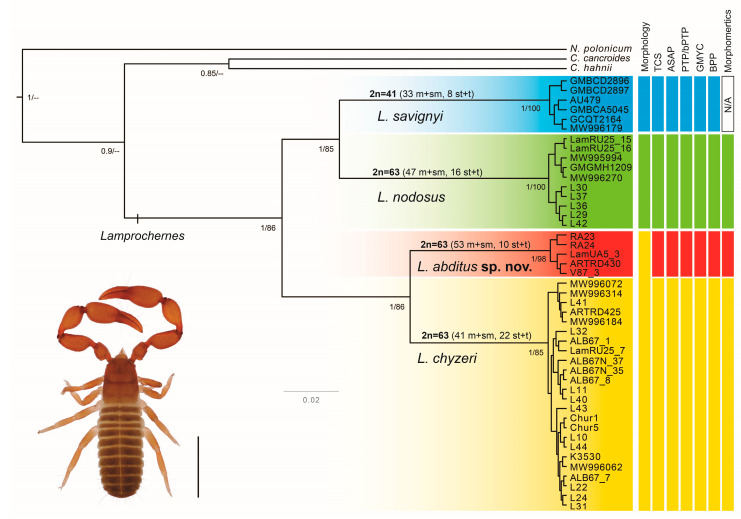
Phylogenetic tree of *Lamprochernes* species with summary of their morphological assignment and the results of different species delimitation approaches (**right**). Topology was obtained in the BI conducted in BEAST. Values on nodes denote support values obtained in both BI and ML analyses (**left** to **right**): Bayesian posterior probabilities (PP), RAxML bootstrap support. Summary of cytogenetic results is depicted above the branches. Abbreviations: m + sm—number of bi-armed chromosomes (metacentric and submetacentric morphology of chromosomes); st + t—number of one-armed chromosomes (subtelocentric and telocentric morphology of chromosomes). **Bottom left** corner insert shows an adult of *L. chyzeri*, scale line: 1 mm.

Both ML (−lnL = 3213.643) and BI analyses recovered four well-supported clades within *Lamprochernes* (Figure 2). The clades corresponded to three *Lamprochernes* species and one undescribed lineage, morphologically undistinguishable from *L. chyzeri*, that we describe as *L. abditus* sp. nov. *Lamprochernes savignyi* was recovered as a sister to *L. nodosus*, while *L. chyzeri* was recovered as sister to *L. abditus* sp. nov. The resulting ML and BI topologies differed mainly in the position of the outgroups (Figure 2, Figure S1 and Figure S2). *Chernes hahnii* was recovered as sister to all *Lamprochernes* diversity in ML analyses, but overall, the relationships among the outgroups were poorly supported in both analyses (Figure 2).

### 3.2. Molecular Species Delimitation and Lamprochernes Haplotype Network

All delimitation approaches applied in the species discovery step unanimously delimited four putative species/independent lineages within the *Lamprochernes* diversity. All approaches correctly assigned an independent status to the three described *Lamprochernes* species and delimited *L. abditus* sp. nov., which was recovered as a sister lineage to *L. chyzeri* in the previous phylogenetic analyses.

**Figure 3:**
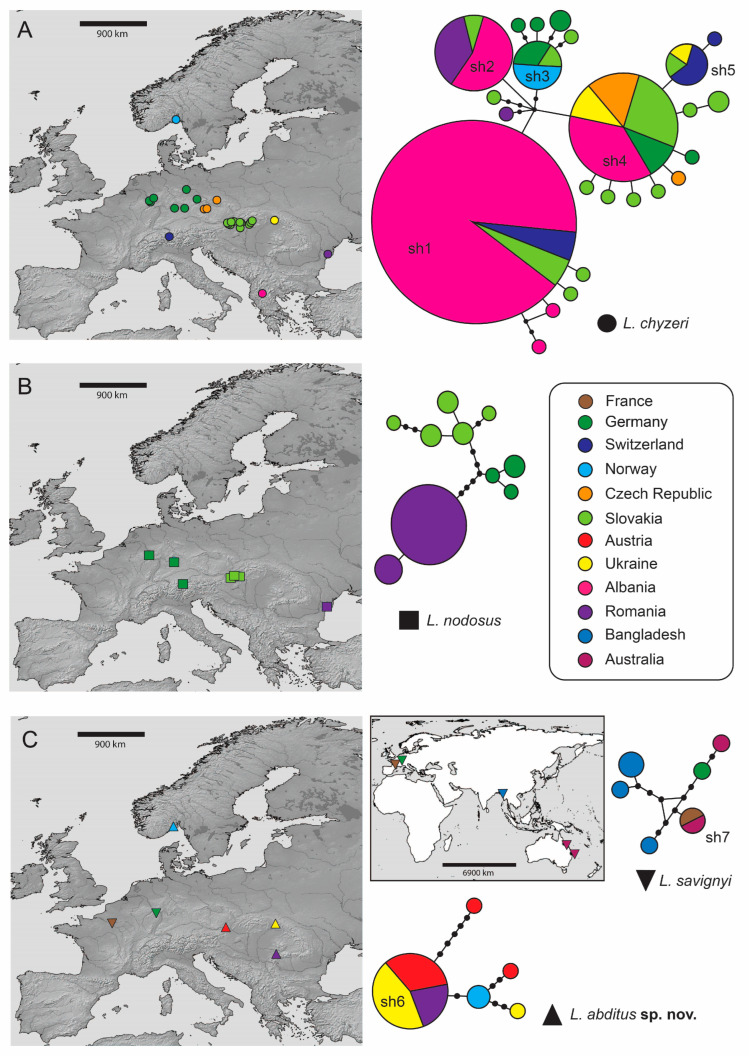
Haplotype networks and sampling locations of *Lamprochernes* species. (**A**) *L. chyzeri* (dots), (**B**) *L. nodosus* (squares), (**C**) *L. savignyi* (inverted triangles) and *L. abditus* sp. nov. (upright triangles). Sampling localities are color coded according to their geographic locations. All maps were created with the help of an online version of SimpleMappr.

Statistical parsimony separated the *Lamprochernes* diversity into four independent networks (Figure 3), based on the 95% parsimony criterion = 10 steps. The size of the network steps in the graphical output was scaled to visualize the haplotype frequency, including collapsed identical haplotypes. The analyses revealed a lack of geographical structure across all four delimited taxa, the same or very similar haplotypes were shared across large geographic distances (Figure 3; Tables S1 and S3). Both PTP and bPTP delimited four species within *Lamprochernes*. The posterior probability (PP) of delimited species inferred in bPTP was as follows: *L. savignyi* = 0.93; *L. nodosus* = 0.81; *L. chyzeri* = 0.95 and *L. abditus* sp. nov. = 0.86. The resulting GMYC delimitation model provided a better fit for the data than the single lineage null model (LR test = 1.180604 e^−9^) and yielded four clusters (confidence interval (CI) 4-4). The four species-model also ranked the highest among the delimited partition schemes in distance based species delimitation performed in ASAP, both with simple *p*-distances (ASAP score = 1, *p* = 5.4 e^−4^) and K80 model (ASAP score = 1, *p* = 1.5 e^−4^).

The species validation step carried out in the BPP confirmed the results of the discovery step. All three runs of the A10 inference recovered all nodes present in the guide tree topology (see Methods) with maximum support (PP = 1). By not collapsing any internal nodes, the analyses favored a four-species delimitation model.

Genetic distances (uncorrelated p-distance and the Tamura-Nei distance) among and within the delimited *Lamprochernes* species are reported in Tables S4 and S5.

### 3.3. Divergence Time Analyses

**Figure 4:**
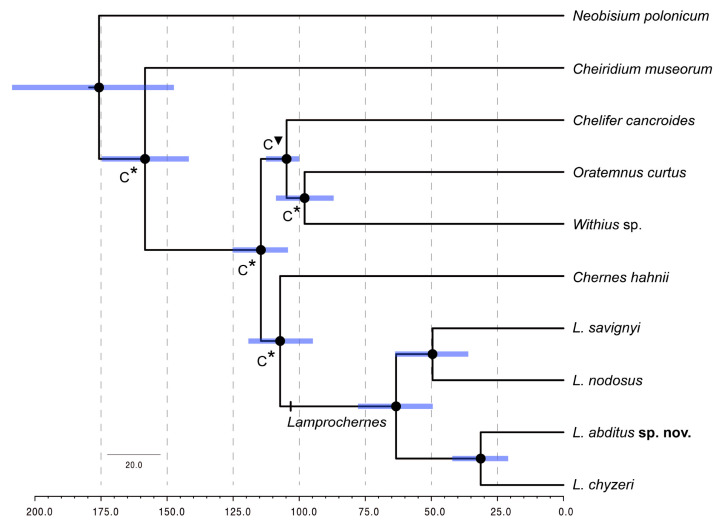
Divergence time estimates of *Lamprochernes* species inferred in BEAST. Dots on the nodes represent support values of PP > 0.95, bars represent the Confidence intervals (CI) of the estimates, C = constrained topology, inverted triangle = fossil calibration, asterisk = calibration from results of Benavides and collaborators [37]. The x-axis represents time in million years.

Divergence time analyses (Figure 4) conducted on “Lampro_time” matrix yielded the following time estimates: Cheliferoidea split was dated to 115 Ma (95% highest posterior density (HDP): 125–104 Ma), Cheliferidae − Atemnidae + Withiidae split approximately to 105 Ma (113–100 Ma). *Lamprochernes* diverged around 107 Ma (119–95 Ma) and started diversifying at 63 Ma (78–49 Ma). *Lamprochernes nodosus* and *L. savignyi* diverged at 49 Ma (63–36 Ma) and *L. chyzeri* and *L. abditus* sp. nov. at 31 Ma (42–21 Ma).

### 3.4. Cytogenetic Analyses

**Figure 5:**
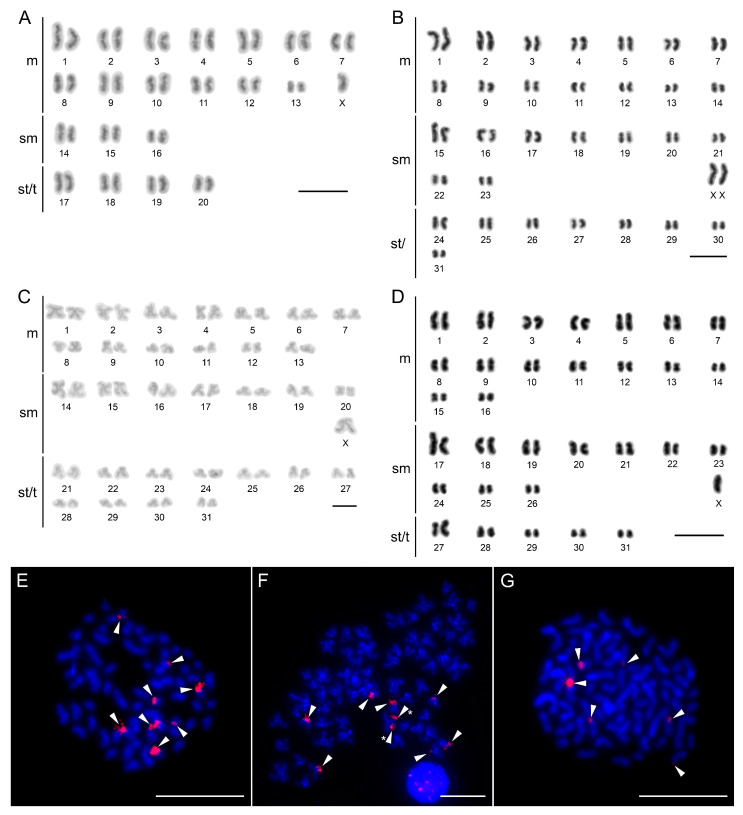
Chromosomes of *Lamprochernes* pseudoscorpions after Giemsa staining (**A**–**D**) and after FISH with 18S rDNA probe (red signals) (**E**–**G**). (**A**) *L*. *savignyi*, male (2n = 41, X0). (**B**) *L. nodosus*, female (2n = 64, XX). (**C**) *L*. *chyzeri*, male (2n = 63, X0). (**D**) *L. abditus* sp. nov., male (2n = 63, X0). (**E**) *L. nodosus*, eight signals of 18S rDNA (arrowheads). (**F**) *L*. *chyzeri*, eight signals of 18S rDNA (arrowheads); arrowheads with asterisks show signals on sister chromatids. (**G**) *L. abditus* sp. nov., six signals of 18S rDNA (arrowheads). Scale lines: 0.01 mm.

The standard chromosome characters were documented in all four *Lamprochernes* species by analyzing meiotic (*L. chyzeri*) or mitotic (*L. nodosus*, *L. savignyi* and *L. abditus* sp. nov.) metaphases (Figure 5). The autosomes in all species gradually decreased in length (Table S6) and all species possessed X0 sex chromosome systems with very large bi-armed X chromosomes. The diploid number was distinctly lower in *L. savignyi* (2n of males = 41, 2n = 42 in females), whereas all three remaining species possessed 2n = 63 in males (2n = 64 in females). Bi-armed chromosomes predominated in karyotypes of all species. *L. savignyi* and *L. abditus* sp. nov. had a four times higher number of bi-armed chromosomes than one-armed ones, while the proportion decreased in *L. nodosus* (three times more bi-armed chromosomes) and *L. chyzeri* (two times more bi-armed chromosomes).

We also identified the number and position of 18S rDNA clusters in *L. chyzeri*, *L. nodosus* and *L. abditus* sp. nov. The signals were found in the terminal positions on the short arms in all species. We observed a different number of signals among analyzed species. *Lamprochernes chyzeri* (Figure 5F) and *L. nodosus* (Figure 5E) possessed signals on eight chromosomes, whereas six signals were observed in *L. abditus* sp. nov. (Figure 5G).

### 3.5. Multivariate Morphometrics

According to the results of stepwise discriminant analyses, the following characters were invariable in the complete dataset of males and were removed from further analyses: leg I trochanter width, leg I femur width, leg I tibia length and leg I tarsus width (all four invariable in *L. abditus* sp. nov.), number of antiaxial accessory teeth fixed finger, leg IV trochanter width and leg IV tarsus width (all three invariable in *L. nodosus*) and palpal hand with pedicel length (invariable in both *L. abditus* sp. nov. and *L. nodosus*). Similarly, several characters were invariable in the (complete) dataset of females: leg I trochanter width (invariable in *L. abditus* sp. nov.), leg IV tarsus width (invariable in *L. nodosus*) and leg I tarsus width (invariable in both *L. abditus* sp. nov. and *L. nodosus*). Correlation coefficients exceeded 0.95 in several character pairs. One character from each pair of highly correlated characters was omitted from the discriminant analyses to avoid bias in the results. From the complete dataset of males, we excluded the length of the palpal patella. From the complete dataset of females, we excluded the length of the palpal patella, length of palpal hand with pedicel, length of palpal chela and length of tibia of leg I.

Stepwise DA of the complete dataset of males and the complete dataset of females identified a set of characters (21 in males and 9 in females, see Table S7) which were most important for the differentiation of the three taxa and were subsequently used to assemble the reduced datasets for further analyses.

Correlation coefficients did not exceed 0.95 in any of the character pairs, all characters were therefore retained for further analyses in the reduced datasets of both males and females. The CDA 1 and 2 and LDA 1 and 2 were computed for the reduced dataset of males and the reduced dataset of females, respectively (see Table S7).

The CDA 1 (with males) and the CDA 2 (with females) yielded very similar results. In both sexes (Figure S3), *L. nodosus* was clearly separated from the other two taxa along the first canonical axis. *Lamprochernes nodosus* was also clearly separated from *L. abditus* sp. nov. along the second axis (Figure S3A) in the case of males, but not in the case of females (Figure S3B). *Lamprochernes abditus* sp. nov. and *L. chyzeri* were clearly separated along the second canonical axis in both males and females. Characters that were most correlated with the canonical axes and thus contributing to the differentiation of particular taxa and clusters are shown in Table S7. The classification functions derived by LDA 1 and LDA 2 can be used for determination of unknown male and female specimens, i.e., their classification into one of the three species (Table S8). Due to the fact that the type specimens of *L*. *chyzeri* and *L*. *mjobergi* could not be used in molecular or cytogenetic analyses, their classification into one of the three species was tested with these functions. All type specimens were classified as *L*. *chyzeri*. 

### 3.6. Morphological Analyses

The results confirmed that *L. nodosus* can be distinguished from *L. abditus* sp. nov. + *L*. *chyzeri* by the round and blunt shape of the protuberance on the palpal trochanter, which is conical and pointed in *L. abditus* sp. nov. + *L*. *chyzeri*. The length of palpal femur and setae number on genital operculae seem to overlap among the species. No new, or additional, characters were found to distinguish the studied species. Detailed morphological descriptions of *L. chyzeri* and *L. nodosus* are provided in “Supplementary Material_Description of *Lamprochernes chyzeri* and *nodosus*” and selected character differences are summarized in Table 1. All raw morphometric data are provided in Table S9.

**Chernetidae Menge, 1855**
**Lamprochernetinae Beier, 1932**
***Lamprochernes* Tömösváry, 1883**

**Figure 6:**
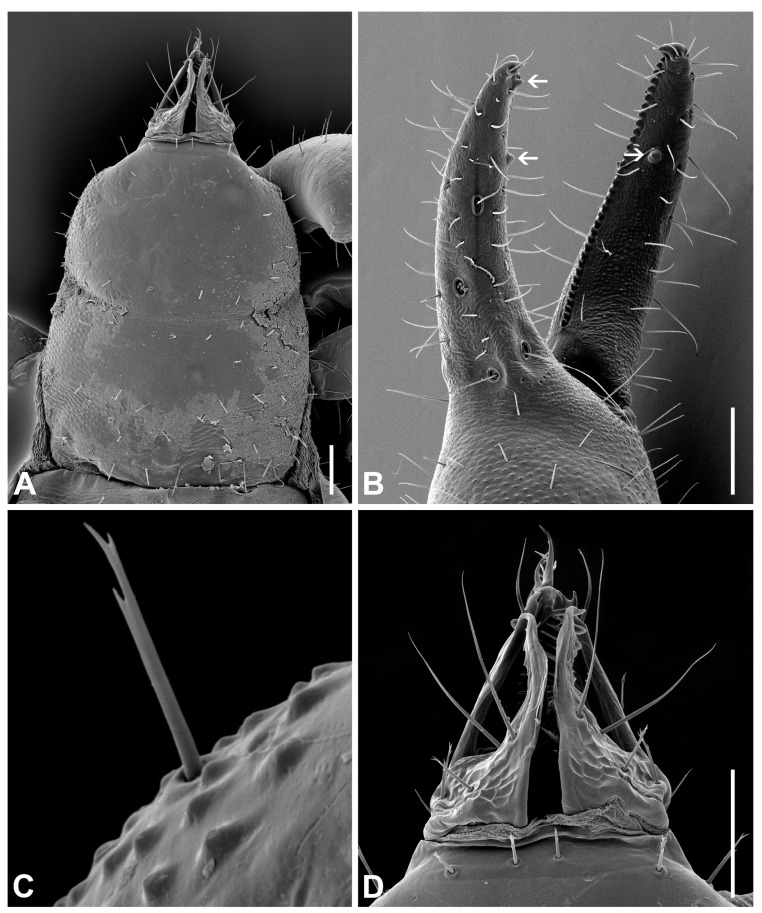
The main diagnostic characters of *Lamprochernes* species (SEM photographs). (**A**) Carapace of *L*. *chyzeri* female. (**B**) Chelal fingers of *L. nodosus* female (arrows point on paraxial teeth). (**C**) Type of pedipalpal seta of *L*. *chyzeri* female. (**D**) Chelicera of *L*. *chyzeri* female. Scale lines: 0.10 mm.

**Figure 7:**
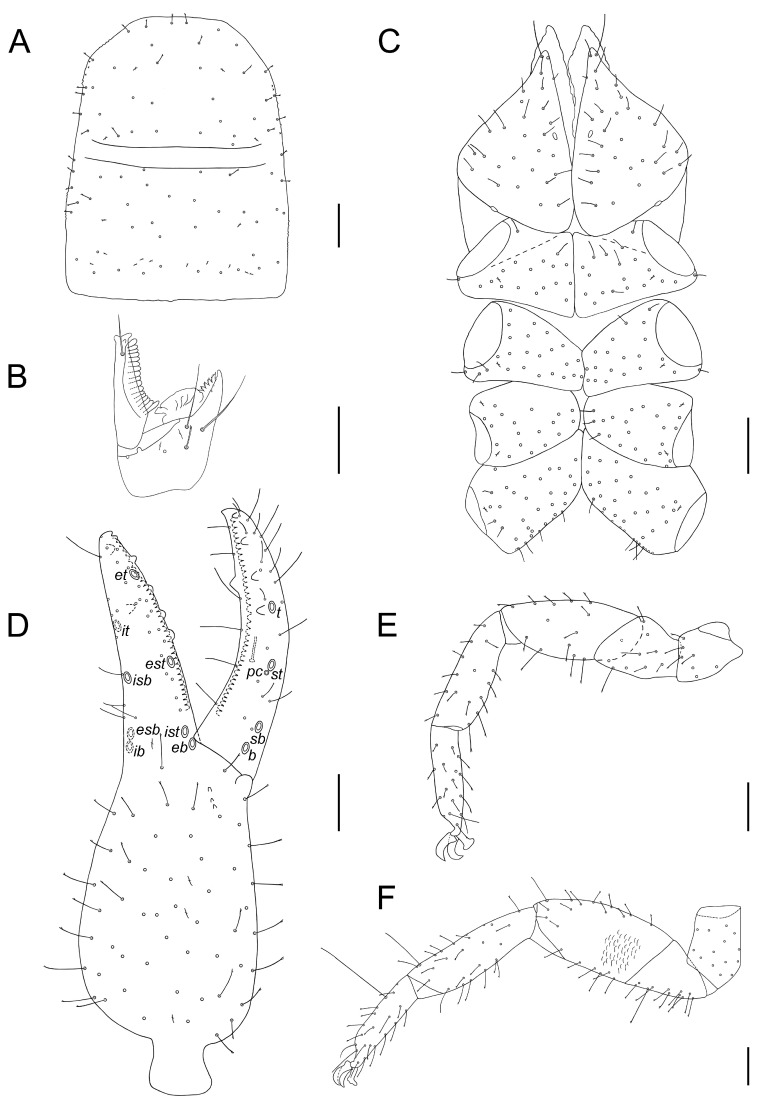
*Lamprochernes abditus* sp. nov. (male holotype: (**A**,**C**); male paratype: (**D**) and female paratype: (**B**,**E**,**F**)). (**A**) Carapace. (**B**) Chelicera (galea broken). (**C**) Coxae. (**D**) Chela with trichobothrial pattern. (**E**) Leg I. (**F**) Leg IV. Scale lines: 0.10 mm. Abbreviations: *b*—basal; *eb*—external basal; *esb*—external sub-basal; *est*—external sub-terminal; *et*—external terminal; *ib*—internal basal; *isb*—internal sub-basal; *ist*—internal sub-terminal; *it*—internal terminal; *pc*—coupled sensillum, *sb*—sub-basal; *st*—sub-terminal; *t*—terminal.

**Figure 8:**
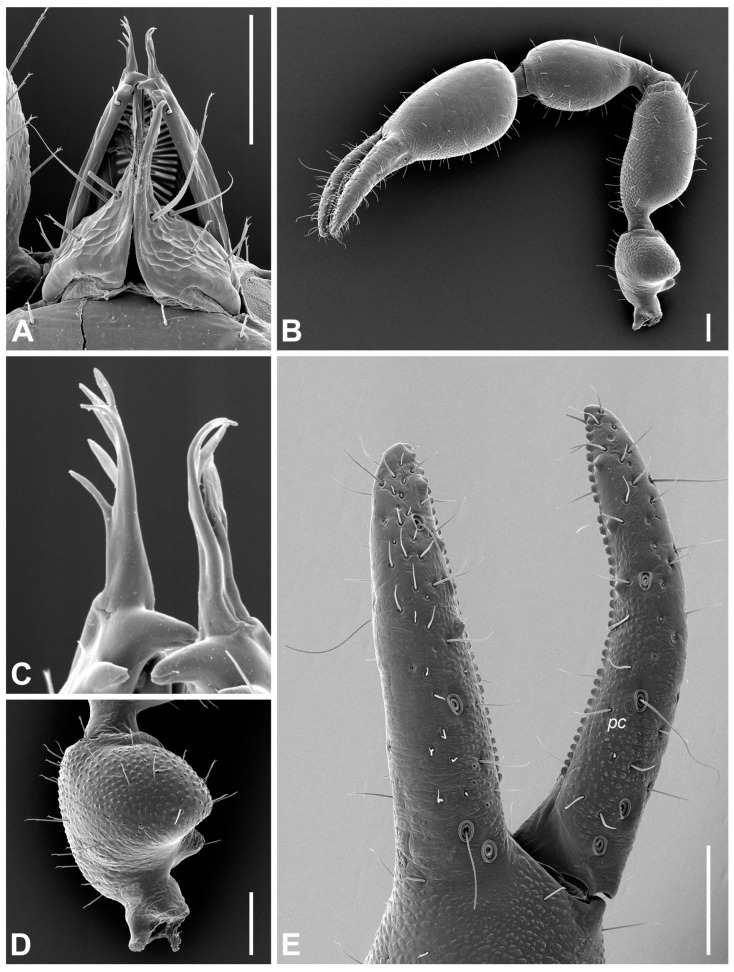
*Lamprochernes abditus* sp. nov., SEM photographs (female paratype: (**A**–**C**,**E**) and male paratype: (**D**)). (**A**) Chelicera. (**B**) Pedipalp. (**C**) Galea. (**D**) Pedipalpal trochanter. (**E**) Chelal fingers. Scale lines: 0.10 mm. Abbreviation: *pc*—coupled sensillum.

**Figure 9:**
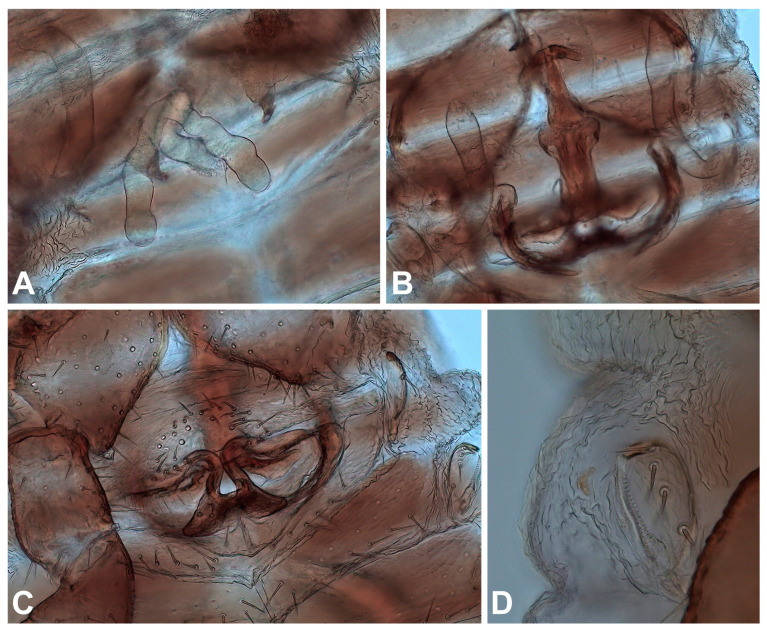
*Lamprochernes abditus* sp. nov., light microscope images (female paratype: (**A**) and male holotype: (**B**–**D**)). (**A**) Genital area of female with spermatheca. (**B**,**C**) Genital area male ((**B**) ventral view, (**C**) dorsal view). (**D**) Detail on pleural membrane and setae on spiracle of holotype male.

**Diagnosis (adults).** Body and pedipalpal setae long, pointed and finely toothed (Figure 6A–D). Carapace (Figure 6A and Figure 7A): longer than broad, almost smooth, epistome absent, anterior margin straight, eyes absent or one indistinct pair present, anterior transverse furrow distinct, posterior one indistinct. Chelicerae (Figure 6D and Figure 7B): small, slightly sclerotized, five setae on hand, one on movable finger; movable finger with slender, well-developed branched galea; rallum of three blades; small, largely unsclerotized teeth situated on both movable and fixed fingers. Pedipalps: chelal fingers with twelve trichobothria (eight on fixed and four on movable chelal finger), venom apparatus developed only in movable chelal finger, fixed chelal finger with two paraxial accessory teeth and movable chelal finger with one paraxial tooth (Figure 6B, Figure 7D and Figure 8E). Tree tactile setae on leg IV: one distally on femoropatella, one distally on tibia and one sub-proximally or sub-medially on tarsus (Figure 7F). Tergite and sternite XI with a pair of long tactile setae. Female spermatheca unpaired, T-shaped (Figure 9A).

***Lamprochernes abditus* sp. nov. (**Figure 7, Figure 8 and Figure 9**)**

**Material examined. Holotype:** 1 ♂ (NHMW-Zoo-AR 29961), Austria, Vienna, Leopoldsberg, 48.27821° N, 16.34720° E, 421 m a.s.l., forest edge, under the bark of a fallen unidentified tree, 17.IV.2015, F. Šťáhlavský leg.

**Paratypes:** 2♀ (NHMW-Zoo-AR 29962, CZ_PK 120/35), 1♂ (CZ_RA 25), Austria, the same data as the holotype; 1♀ (NHMW-Zoo-AR 29963), Austria, Vienna, Leopoldsberg, 48.27838° N, 16.34595° E, 433 m a.s.l., forest edge, under the bark of a fallen unidentified tree, 21.IV.2014, F. Šťáhlavský and I. Hynková leg.; 2♀ (NHMW-Zoo-AR 29964, CZ_PK 92/35), Romania, Poiana Mărului, 45.42820° N, 22.46638° E, 523 m a.s.l., near the road, under the bark of an unidentified fruit tree, 17.VI.2016, J. Christophoryová leg.; 3♂ , 2♀ (2♂ , 2♀ NHMW-Zoo-AR 29965–29968; 1♂ CZ_PK 94/35), Ukraine, Nevyts’ke, 48.68274° N, 22.40513° E, 132 m a.s.l., meadow near the river, under the bark of *Populus* sp., 8.VI.2016, J. Christophoryová and D. Jablonski leg.

**Diagnosis**

*Lamprochernes abditus* sp. nov. can be distinguished from *L*. *muscivorus* Redikorzev, 1949, *L. nodosus* and *L. savignyi* by the shape of protuberance of palpal trochanter (in *L. abditus* sp. nov. conical and pointed one vs. in *L*. *muscivorus*, *L. nodosus* and *L. savignyi* blunt and rounded one [64,68,115]). The difference between *L. abditus* sp. nov. and *L*. *moreoticus* (Beier, 1929) is in the trichobothrial pattern on the movable chelal finger (in *L. abditus* sp. nov. sub-terminal trichobothrium situated between terminal and sub-basal trichobothria vs. in *L*. *moreoticus* sub-terminal trichobothrium situated closer to sub-basal trichobothrium) [64]. *Lamprochernes foxi* (Chamberlin, 1952) was recorded in the USA and *L*. *minor* Hoff, 1949 in Canada, Turkey and USA with no distribution data in Europe [34]. *Lamprochernes abditus* sp. nov. differs from *L*. *foxi* by terminating venom apparatus in nodus ramosus in the movable chelal finger (in *L. abditus* sp. nov. close to sub-terminal trichobothrium vs. in *L*. *foxi* between terminal and sub-terminal trichobothria) [116]. *Lamprochernes minor* can be distinguished from *L. abditus* sp. nov. by the S-shaped palpal femur and strongly granulated carapace (not present in *L. abditus* sp. nov.) [117]. To distinguish *L. abditus* sp. nov. from *L*. *leptaleus* and *L*. *procer* (Simon, 1878) is more complicated. Their descriptions are rudimentary, both species were questioned previously by Beier [64] and require a revision. Based on the available published data, *L. abditus* sp. nov. can be distinguished from *L*. *procer* by its smaller body size (in *L. abditus* sp. nov. 1.95–3.08 mm vs. in *L*. *procer* 4.2 mm) [64]. The diagnosis of *L*. *leptaleus* is controversial, while the species barely possesses the main diagnostic genus characters [65].

Morphologically *L. abditus* sp. nov. closely resembles *L. chyzeri*, from which it cannot be distinguished by basic morphological characters used in Chernetidae systematics. *Lamprochernes abditus* sp. nov. and *L. chyzeri* can be distinguished by multivariate analyses of morphologic and morphometric characters in males as follows: setae number on anterior and posterior disk of the carapace, setae number on tergite VI and sternite X, palpal trochanter width, leg I trochanter length and leg I patella width; in females: leg IV tarsus length (Table S7).

Molecularly, *L. abditus* sp. nov. can be distinguished from hereby analyzed *Lamprochernes* species by the following unique nucleotide substitutions in the animal DNA barcode sequence alignment (Supplementary Material_Alignment): A (13), A (39), G (60), (A) 99, C (141), T (165), C (202), T (204), C (219), A (282), C (315), T (330), C (345), G (354), G (369), C (414), A (432), G (477), T (504), A (510), A (555), A (597). *Lamprochernes abditus* sp. nov. can be further distinguished from *L. chyzeri* by the following unique nucleotide substitutions in the animal DNA barcode sequence alignment: T (21), C (23), A (27), A (39), A (42), G (60), C (73), T (78), T (81), A (84), T (90), A (99), A (105), T (114), A (135), C (141), G (159), T (165), T (180), A (195), C (202), T (204), A (207), C (216), C (219), T (237), G (240), T (273), A (282), A (291), A (309), C (315), A (318), A (321), T (330), C (345), G (354), T (363), G (369), T (372), T (375), A (378), A (408), C (414), A (432), G (477), A (486), T (504), G (507), A (510), A (531), A (549), G (552), G (555), T (585), T (597).

Cytogenetically *L. abditus* sp. nov. can be also distinguished from *L. chyzeri* and *L. nodosus*. All three species possess 2n = 63 in males; however, *L. abditus* sp. nov. has a four times higher number of bi-armed chromosomes than one-armed ones, while *L. nodosus* possesses only three times more bi-armed chromosomes and *L. chyzeri* only two times more bi-armed chromosomes.

**Etymology.** The specific name is derived from the Latin word “*abditus*”, meaning hidden. The species cannot be distinguished from *L. chyzeri* by standard morphological characters. In order to identify *L. abditus* sp. nov., complex morphometric analyses, molecular or cytogenetic approaches are necessary.

**Description. Adult male (4** **♂) and female (7** **♀).**

All measurements and ratios for both sexes are summarized in Tables S2 and S9. Body surface generally smooth, lateral margins of carapace and internal margins of pedipalpal trochanter, femur, tibia and chela moderately granulate (Figure 8B). Body and pedipalpal setae long, pointed and finely toothed (Figure 7A,D and Figure 8B,D). **Carapace** (Figure 7A): more long than broad, almost smooth; anterior transverse furrow distinct, posterior one indistinct; epistome absent, anterior margin straight; eyes absent; carapace with ♂ 73–87 setae (♀ 75–107), ♂ 34–41 (♀ 39–53) of them on anterior disk, ♂ 26–28 (♀ 24–37) on medial disk, posterior margin with ♂ 12–18 setae (♀ 12–17); carapace with six macrolyrifissures: two pairs situated on anterior disk and one pair located on medial disk, posterior margin with ten microlyrifissures (Figure 7A). **Opisthosoma:** Tergites I–X divided, XI partly divided. Chaetotaxy of tergites I–XI ♂: 16–19 (left hemitergite 7–10 + right hemitergite 8–9): 16–18 (8–9 + 8–9): 15–17 (7–8 + 7–9): 18–20 (9–11 + 9): 19–22 (9–11 + 9–11): 18–21 (9–11 + 9–10): 18–23 (9–12 + 9–11): 19–21 (9–10 + 10–11): 18–21 (9–11 + 9–10): 17–21 (8–11 + 9–10), tergite XI with 10 setae (5 + 5) and with a pair of long tactile setae. Chaetotaxy of tergites I–XI ♀: 17–19 (left hemitergite 8–10 + right hemitergite 8–9): 16–19 (8–10 + 8–9): 15–16 (7–8 + 7–8): 18–21 (8–11 + 9–10): 19–23 (10–11 + 9–12): 21–23 (11–12 + 10–12): 20–24 (10–12 + 9–12): 21–24 (10–12 + 10–13): 20–26 (10–13 + 10–13): 18–23 (8–12 + 9–11), tergite XI with 10–12 setae (5–6 + 5–6) and with a pair of long tactile setae. Sternites I–X divided, XI partly divided. Chaetotaxy of sternites IV–XI ♂: 17–19 (left hemisternite 8–9 + right hemisternite 8–10): 25–30 (12–16 + 12–14): 25–29 (12–15 + 13–14): 25–30 (12–14 + 13–16): 24–28 (12–13 + 12–15): 24–28 (12–15 + 12–14): 18–24 (9–12 + 9–12), sternite XI with 10 (5 + 5) and with a pair of long tactile setae. Chaetotaxy of sternites IV–XI ♀: 8–12 (left hemisternite 4–6: right hemisternite 4–6): 26–32 (13–15 + 13–18): 29–37 (15–20 + 13–17): 25–36 (13–18 + 12–18): 26–37 (13–19 + 13–18): 25–33 (13–17 + 12–16): 20–27 (10–12 + 10–15), sternite XI with 10–12 (5–6 + 5–6) and with a pair of long tactile setae. Spiracles: ♂ sternite III with four setae (♀ 3 setae), sternite IV with four setae (♀ 4 setae) (Figure 9D). Anterior genital operculum ♂ with 29–37 setae and two lyrifissures, posterior operculum with 14–18 setae and two lyrifissures (Figure 9C). For male internal genitalia see Figure 9B,C. Anterior genital operculum ♀ with 19–32 setae and one or two lyrifissures and posterior operculum with 9–15 setae and two lyrifissures. For female spermatheca see Figure 9A. Pleural membrane longitudinally striate (Figure 9D). **Chelicera** (Figure 7B and Figure 8A,C): small, slightly sclerotized; hand with five setae and two lyrifissures, one seta on movable finger; movable finger with slender, well-developed branched galea, ♂ with 5 terminal rami, ♀ with 5–6 terminal rami; serrula exterior with 17–18 blades; rallum of three blades; small, largely unsclerotized teeth situated on both movable and fixed fingers (Figure 7B). **Coxae** (Figure 7C): ♂ pedipalpal coxa excluding manducatory process with 24–26 setae, manducatory process with 3 setae and 1 microseta; coxal chaetotaxy of legs I–IV: 18–19: 20–24: 18–22: 34–36, all setae acuminate; lyrifissures: none on pedipalpal coxa, one on each pedal coxa I, II and IV and two on each pedal coxa III; each pedipalpal coxa with two maxillary lyrifissures (Figure 7C). ♀ pedipalpal coxa excluding manducatory process with 21–26 setae, manducatory process with 3 setae and 1 microseta; coxal chaetotaxy of legs I–IV: 21–23: 23–26: 21–22: 38–42, all setae acuminate; lyrifissures: none on pedipalpal coxa, one on each pedal coxa I–IV; each pedipalpal coxa with two maxillary lyrifissures. **Pedipalp** (Figure 7D and Figure 8B,D,E): for granulation and shape of pedipalpal segments see Figure 8B; protuberance on pedipalpal trochanter conical and pointed (Figure 8B,D); femur abruptly pedicellate; pedipalpal hand with three lyrifissures, one on the base of fixed chelal finger (Figure 7D and Figure 8E); two lyrifissures on pedipalpal patella (Figure 8B). Chelal fingers with 12 trichobothria (eight on fixed and four on movable chelal finger); fixed finger with trichobothrium *it* closer to *isb* than to tip of fixed finger, *est* slightly distal to *isb*, *esb*–*ib* and *ist*–*eb* at about same level (Figure 7D). Movable chelal finger with trichobothrium *st* situated slightly closer to *t* than to *sb*, trichobothrium *sb* closer to *b* than to *st* (Figure 7D and Figure 8E). Venom apparatus developed in movable chelal finger terminating in nodus ramosus close to sub-terminal trichobothrium (Figure 7D); one coupled sensillum *pc* situated near *st* (Figure 7D and Figure 8E); fixed chelal finger with ♂ 32–35 (♀ 30–35) and movable chelal finger with ♂ 34–37 (♀ 32–35) marginal teeth; fixed chelal finger with 5–6 antiaxial and movable chelal finger with 4–5 antiaxial accessory teeth (Figure 8E); fixed chelal finger in both sexes with two paraxial accessory teeth and movable chelal finger with one paraxial tooth (Figure 7D). **Legs** (Figure 7E,F): all claws of legs smooth, arolia simple and shorter than claws. Leg IV with three tactile setae: one distally on femoropatella, one distally on tibia and one sub-proximally on tarsus (Figure 7F).

## 4. Discussion

### 4.1. Species Delimitation in Lamprochernes

Barcoding has helped to uncover cryptic diversity among various arachnid groups, including pseudoscorpions [29,118,119,120,121]. The results of this study highlight the usefulness of barcoding data for cryptic-diversity detection, even in regions considered as well known. Our molecular species delimitation analyses delimited four independent lineages within our dataset; three corresponding to nominal *Lamprochernes* species (*L. chyzeri*, *L. nodosus* and *L. savignyi*) and one additional lineage *L. abditus* sp. nov., which cannot be distinguished from *L. chyzeri* by standard morphological characters used in Chernetidae taxonomy. Single locus delimitation methods have tendencies to over-split the taxa and different approaches often disagree on the number of delimited units [122,123]. In our case, the molecular approaches implemented in the species discovery step yielded the same outcome (i.e., four delimited lineages) that was also congruent with the results of the species validation based on molecular data conducted in BPP.

The distance analyses subsequently uncovered significant divergences among the delimited *Lamprochernes* lineages (p-distance: 11.1–17.8%, Tamura-Nei: 12.2–20.1%). Interestingly, *Lamprochernes abditus* sp. nov. and *L. chyzeri*, indistinguishable morphologically, differed by 11.1–12.2% (p-distance and Tamura-Nei, respectively). Such distances are more congruent with interspecific divergences among valid pseudoscorpion species than with reported values of intraspecific diversity [29,118,119]. The intraspecific distances reported from other Chernetidae taxa inhabiting the same geographic region are generally much lower (~1–5.5%), with the exception of *Dinocheirus panzeri* (C.L. Koch, 1836) that reached 12.82% and most likely harbors cryptic diversity [29]. Moreover, if *L. abditus* sp. nov. and *L. chyzeri* were considered as a single species, its intraspecific distances would seem to be particularly high in comparison to the values reported from other phoretic pseudoscorpions that were studied in detail. Genetic distances separating two lineages, potentially representing cryptic species, detected within *Chernes hahnii*, were less than 5% [43]. In *Dinocheirus arizonensis* (Banks, 1901), the main clades referred to as “independent lineages” differed only by 2.6% [40].

Cryptic species detected by molecular methods often remain undescribed due to the lack of species validation by other approaches [10,124]. In our *Lamprochernes* delimitation, we also implemented morphological, morphometric and cytogenetic analyses, in order to verify the delimited species and to avoid taxa over-splitting. Morphological characters used in *Lamprochernes* systematics can be difficult to interpret. For example, the shape of palpal trochanter protuberance, one of the main characters distinguishing *L. chyzeri* and *L. nodosus*, is challenging to categorize either as “blunt and rounded” or “conical and pointed”, especially when the sample size is small. The identification key [69] mentions the length of the palpal femur as an additional character, but the values for the two species may also overlap. The results of our analyses show that even though the values overlap, the length of the palpal femur in *L. nodosus* is generally shorter (range from 0.36–0.50 mm) than in *L*. *chyzeri* (range from 0.44–0.65 mm) and the character could be used as diagnostic in combination with the shape of palpal trochanter protuberance. The last and the most confusing character was the number of setae on genital operculum in females. According to the key in Legg and Jones [36], anterior genital operculum bears more than 20 setae in *L. nodosus* and 9–11 setae in *L*. *chyzeri*; however, in the detailed description they mentioned 30–34 setae in *L*. *chyzeri*. Our results show that the values of setae numbers on genital operculae in both sexes of both species slightly overlap (in *L. nodosus*: ♂ anterior genital operculum with 18–23 setae, posterior one with 9–12; ♀ anterior genital operculum with 17–22 setae, posterior one with 6–9 setae vs. in *L*. *chyzeri*: ♂ anterior genital operculum with 27–41 setae, posterior one with 11–19 setae; ♀ anterior genital operculum with 21–29 setae, posterior one with 9–15 setae) but in general this character could be applied to distinguish *L. nodosus* from *L*. *chyzeri* in combination with the above mentioned characters. However, neither of these characters could be used for separating *L. abditus* sp. nov. from *L. chyzeri*.

We employed morphometric analyses to complement our morphology-based species validation step [125]. Morphometric approaches were successfully used in pseudoscorpion taxonomy to distinguish *Ephippiochthonius* Beier, 1930 (Chthoniidae) [46] and *Apolpium* Chamberlin, 1930 (Olpiidae) [126] species. In Chernetidae, multivariate morphometric techniques successfully detected morphological differentiation of three *Lasiochernes* Beier, 1932 species and highlighted the most reliable characters for their identification [47]. Our results suggest that multivariate morphometrics can be used to distinguish analyzed *Lamprochernes* species, including the cryptic *L. abditus* sp. nov.

Our results suggest that multivariate morphometrics can be used to distinguish analyzed *Lamprochernes* species, including the cryptic *L. abditus* sp. nov. Some of the characters that were the most correlated with the canonical axes, i.e., characters contributing to the differentiation of *L. nodosus* from *L. abditus* sp. nov. and *L*. *chyzeri* were the same for both sexes (e.g., setae number on posterior disk of carapace, palpal femur length). The most correlated character contributing to the differentiation of *L. abditus* sp. nov. from *L*. *chyzeri* was for both of the sexes length of tarsus of leg IV. The importance of palpal femur length (in both sexes) and setae number on genital opercula (anterior only in males, posterior in both sexes) as characters traditionally used to distinguish *L. nodosus* and *L*. *chyzeri* [36,47,69] was also confirmed by the results of multivariate morphometrics. The robustness of the inference derived from the morphometric analyses used in our study might be limited by the low number of analyzed individuals, especially *L. abditus*. The studied type specimens of *L*. *chyzeri* and *L*. *mjobergi* tested by classification functions derived by LDA analyses were classified into *L*. *chyzeri*. However, the identity of material of *L*. *chyzeri* deposited in other collections is still unclear and further revision is needed.

Differences in karyotypes among closely related taxa are useful for taxonomic purposes. Cytogenetic methods are commonly implemented in arthropod systematics only among cytogenetically well-understood groups such as Orthoptera [127], Lepidoptera [128] and Diptera [129], but are still underappreciated in species delimitation of arachnids. Karyotype variability previously verified the species status among taxa with little morphological differentiation in scorpions [130], spiders [131] and pseudoscorpions [54]. However, cytogenetic analyses are rarely implemented as a species validation step in the integrative species delimitation pipeline [31,132]. Our results, particularly those related to 2n interspecific variability, thus highlight the importance of incorporating the cytogenetic data in pseudoscorpion diversity research. We identified a significantly different 2n in *L. savignyi* compared to the remaining *Lamprochernes* species. Additionally, differences in chromosomal morphology were detected among the species with the same 2n (*L. nodosus*, *L. chyzeri* and *L. abditus* sp. nov.). Similarly, karyotype differences have suggested the presence of cryptic species among taxa of almost all pseudoscorpion families [50,52,53]; however, these differences were never used for species delimitation with exception of *Roncus montsenyensis* Zaragoza and Šťáhlavský, 2008 (Neobisiidae) [54].

Additionally, the implementation of molecular cytogenetic techniques (FISH) detected differences in numbers of 18S rDNA clusters among *L. nodosus*, *L. chyzeri* (eight clusters) and *L. abditus* sp. nov. (six clusters). The differences may indicate early differentiation between *L. abditus* sp. nov. and *L. chyzeri* that cannot be identified using conventional staining. Unfortunately, we have limited knowledge concerning 18S rDNA clusters in pseudoscorpions. The only available data proceed from *Neobisium slovacum* Gulička, 1977 (Neobisiidae) possesses only two pairs of these clusters [133]. The most frequent number of 18S rDNA in arthropods is one pair [134]. The higher number of 18S rDNA clusters in both *Lamprochernes* and *Neobisium* may thus be a consequence of genome duplication in the whole Arachnopulmonata clade [135]. However, this hypothesis needs to be tested in more species.

### 4.2. Morphological Stasis in Lamprochernes Evolution

Species crypsis is often perceived as a temporary state, indicating that distinguishing morphological characters among cryptic species have not yet been identified either due to lack of scientific effort or character-bias interpretation [12,124]. An alternative view is that the taxa in question have not yet accrued morphological differences after recent diversification [1]. According to the results of the divergence time analyses, *L. abditus* sp. nov. diverged from *L. chyzeri* during the Oligocene, approximately 31 Ma, which exceeds the ages of putative cryptic species reported from other pseudoscorpion taxa. For example, the divergence between the two clades of *C. hahnii* was dated to the Pleistocene, likely linking the split to the quaternary climatic oscillations in Europe [43] and the diversification within Australian *Pseudotyrannochthonius giganteus* Beier, 1971 species complex spanned from the late Miocene to the Pliocene [136]. Pseudoscorpions possess an accelerated substitution rate [137], which may skew the divergence times when the general arthropod mitochondrial substitution rate is implemented. We therefore used fossil record (age of Cheliferidae) combined with ages inferred by Benavides and collaborators [37], proceeding from transcriptomic data, dense taxon sampling and relatively rich fossil records, to calibrate our divergence time analyses and obtain a more reliable outcome. Our analyses yielded time estimates similar to those obtained by Benavides and collaborators [37] in their relaxed log normal clock implementation. The divergences within Cheliferoidea such as basal Cheliferoidea split (~115 Ma), age of Cheliferidae (~105 Ma) and Chernetidae diversification (~107 Ma) are comparable between the analyses (~128 Ma, 95 Ma, 101 Ma, respectively [37]), indicating that our divergence time estimates hypothesis is also reliable in terms of the inferred ages of *Lamprochernes* species.

Ancient ages of cryptic species were reported from both vertebrate [138] and invertebrate taxa [139]. The reasons leading to such morphological uniformity may be related to particular habitat conditions and niche conservatism [140], or to a sedentary lifestyle and allopatric evolution with less pressure for morphological differentiation [141]. An extreme case of morphological stasis, with divergence time among taxa between ~275 Ma to 18 Ma, has been recently reported e.g., from annelids [140]. The evolutionary pressure to evolve morphological differences should be presumably stronger for species that evolved in sympatry, or for those that entered in a secondary contact; that is, unless the mating partner recognition is not based on morphological appearance [142]. The genus *Lamprochernes* (and the whole family Chernetidae) has reduced eyes [102], indicating that something other than visual cues must be involved. *Lamprochernes abditus* sp. nov. and *L. chyzeri* overlap geographically and both can be found in the same types of habitat, suggesting that a reproductive isolation mechanism exists between the species. Conspecific recognition in pseudoscorpions may be facilitated by a sophisticated chemo- and mechanoreceptor network located in the pedipals [143]. Additionally, a postzygotic reproductive barrier may have already evolved between *L. abditus* sp. nov. and *L. chyzeri.* Such a mechanism was detected at 11% divergence among populations of another chernetid pseudoscorpion *Cordylochernes scorpioides* (Linnaeus, 1758) [38,144].

### 4.3. Distribution and Dispersal Capabilities of Lamprochernes

All *Lamprochernes* species analyzed in this study are found in the same types of habitats (e.g., in decaying material such as compost heaps, manure heaps and under tree bark) and likely have at least partially overlapping distributions in Europe. The genus uses phoresy in its dispersal [145], which is a known adaptation to inhabiting temporary habitats such as decaying plant material [146]. This manner of dispersal appears to be very efficient among the analyzed species, with the exception of *L. nodosus*. *Lamprochernes abditus* sp. nov., *L. chyzeri* and *L. savignyi* completely lack geographic structuring in their genetic background. Identical haplotypes can be commonly found across geographically distant localities. In *L. chyzeri*, “shared haplotype 4” was present at localities in Germany, Czech Republic, Slovakia, Ukraine and Albania up to 1519 km apart. Identical haplotypes were also detected in *L. abditus* sp. nov. and *L. savignyi*. A similar lack of genetic structuring across a comparable geographic scale was previously found in *C. hahnii*. This species shows all the signs of phoretic dispersal (e.g., inhabiting temporary habitats, lack of genetic structuring), but unlike *Lamprochernes*, it was never directly collected on a carrier host [43]. An extreme case of haplotype sharing can be found in *L. savignyi*, where “shared haplotype 7” was detected in France and also from two localities in Australia (16,678 km apart). Although there is some evidence that pseudoscorpions may also be phoretic on birds [147,148], the fact that *Lamprochernes* is commonly found in compost heaps, including those associated with botanical gardens, accidental human introduction cannot be ruled out [62]. The only species showing geographic structuring among its populations is *L. nodosus.* Such a difference could be explained by the species’ affinities to a host that is a weaker flyer, or it is associated with very particular and patchy habitats [40]. More data, including field observations, is needed to confirm this hypothesis.

Prior to this study, *L. chyzeri* appeared to be less common [34], but the lack of its records could have been caused by its misidentification for *L. nodosus*. Thanks to our sampling efforts and the data in public databases (Genbank, BOLD), we were able to expand the species’ distributions by detecting incorrectly identified specimens of *L. chyzeri* (identified as *L. nodosus*) and *L. abditus* sp. nov. (identified as *L*. *chyzeri*) (Table S1). *Lamprochernes chyzeri* is recorded for the first time from Ukraine and *L*. *savignyi* from Bangladesh and France (see Table S1, Figure 3). Our field data also further suggest that the genus can be easily collected from compost and manure heaps instead of from under tree bark, especially when more specimens are needed from one locality [69]. The genus *Lamprochernes* represents an excellent model system for evolutionary research; our findings thus improve both sampling methodology and species identification for future studies.

## 5. Conclusions

We implemented an integrative approach combining molecular, cytogenetic and morphological data for species delimitation in European populations of the genus *Lamprochernes* and detected an ancient cryptic lineage within its diversity. *Lamprochernes abditus* sp. nov. is indistinguishable from *L*. *chyzeri* by standard morphological features used in pseudoscorpion taxonomy. However, it differs significantly from all the analyzed taxa by molecular, cytogenetic, morphometric and morphologic characters by multivariate analyses. Most *Lamprochernes* species commonly shared haplotypes across geographically distant populations, which suggests phoretic dispersal is highly efficient in this group.

## Figures and Tables

**Table 1:** Synopsis of the diagnostic characteristics to differentiate four compared species. New observed data in combination with the published data [36,56,62,64,70,110,111,112,113,114]. Values of measured characters are in mm.

Character/Species	*L. abditus* sp. nov.	*L*. *chyzeri*	*L. nodosus*	*L*. *savignyi*
Shape of palpal trochanter protuberance	pointed	pointed	rounded	rounded
Palpal chela, length	0.92–1.02	0.77–1.04	0.71–0.91	0.69–0.85
Palpal femur, length	0.54–0.59	0.44–0.65	0.36–0.50	0.32–0.46
Setae number on male genital operculum: anterior/posterior	29–37/14–18	27–41/11–19	18–23/9–12	15–22/10–14
Setae number on female genital operculum: anterior/posterior	19–32/9–15	21–29/9–15	17–22/6–9	16–21/5–8
Tarsus of leg IV length/distance of the tactile seta from the tarsus base, ratio	2.71–3.29	2.88–3.54	2.72–3.68	2.40–2.77
Distribution	Austria, Norway, Ukraine, Romania	Europe, Kazakhstan, Russia, Turkey	Europe, Central Africa, South Asia	Africa, Australia, America, New Zealand, South Asia, West to Switzerland and South Europe

## Data Availability

The data presented in the study are available in the article.

## Supplementary Materials

The following supporting information can be downloaded at: https://www.mdpi.com/article/10.3390/insects14020122/s1. Figure S1: Tree topology obtained in ML analyses conducted in RAxML; Figure S2: Tree topology obtained in BI conducted in BEAST; Figure S3: Canonical discriminant analyses of the (reduced) datasets containing only the most important differentiating characters; Table S1: Sampled pseudoscorpion material; Table S2: Descriptive statistics of measured morphological characters of the studied *Lamprochernes* species; Table S3: Geographical structure across four delimited taxa; Table S4: Uncorrected p-distances, Tamura—Nei genetic distances within *Lamprochernes* species; Table S5: Uncorrected p-distances, Tamura—Nei genetic distances among *Lamprochernes* species; Table S6: Measurements of the relative diploid set length (%DSL) and arm ratio (AR) of chromosomes including standard deviation (±SD) of *Lamprochernes* species; Table S7: The list of the analyzed characters and the results of the canonical discriminant analyses CDA 1 and CDA 2; Table S8: Classification functions for males and females of *Lamprochernes abditus* sp. nov., *L*. *chyzeri* and *L. nodosus* derived from linear classificatory discriminant analyses (LDA 1 and LDA 2 respectively); Table S9: Raw morphometric data of studied *Lamprochernes* species. Other Supplementary files: Supplementary Material_Description of *Lamprochernes chyzeri* and *L. nodosus.*; Supplementary Material_Alignment.
