# Supplementary material for: Integrative Taxonomy Approach Reveals Cryptic Diversity within the Phoretic Pseudoscorpion Genus Lamprochernes (Pseudoscorpiones: Chernetidae)
Source: Insects. 2023 Jan 25;14(2):122. doi: 10.3390/insects14020122 (PMC9964657; doi:10.3390/insects14020122)
Supplement: Supplementary file 1 [file insects-14-00122-s001.zip › Supplementary Material_Description of Lamprochernes chyzeri and nodosus.pdf]

Supplementary Material_Description of *Lamprochernes chyzeri* and *nodosus*

Lamprochernes chyzeri

Material examined:

Types:

Syntypes of *Lamprochernes chyzeri*: HNHM Pseudoscorp-392: 5 males, 1 female (for details see Tables S1 and S9).

Lamprochernes mjobergi (Tullgren, 1909) (synonymised by Beier [68]): NHRS-TOBI000005234: 1 female (for details see Table S1).

Other material: For details see Tables S1 and S9.

Males (66 specimens analysed) (Table S2). Chaetotaxy of carapace: 69–95 setae, 35–50 of them on anterior disk, 22–37 on medial disk, posterior margin with 10–15 setae. Cheliceral galea with 5–6 short terminal rami, serrula exterior with 17–18 blades. Pedipalpal coxa excluding manducatory process with 19–24 setae, manducatory process with 3 setae and 1 microseta; coxal chaetotaxy of legs I–IV: 15–18: 18–21: 16–22: 17–34. Lyrifissures: none on pedipalpal coxa, one on each pedal coxa II–IV and one to three on pedal coxa I; each pedipalpal coxa with two maxillary lyrifissures. Palps: fixed chelal finger with 29–36 and movable chelal finger with 31–38 marginal teeth; fixed chelal finger with 4–6 and movable chelal finger with 3–5 antiaxial accessory teeth. Chaetotaxy of tergites I–XI: 14–20 (left hemitergite 7–10 + right hemitergite 6–11): 15–22 (7–10 + 7–12): 12–19 (6–10 + 5–10): 16–26 (8–13 + 8–13): 18–27 (9–13 + 9–14): 19–30 (9–15 + 9–15): 18–27 (9–14 + 9–14): 18–26 (9–13 + 9–14): 18–27 (9–14 + 8–13): 16–26 (9–13 + 7–13), tergite XI with 8–12 setae (4–6 + 4–6) and with a pair of long tactile setae. Chaetotaxy of sternites IV–XI: 14–25 (left hemisternite 7–12 + right hemisternite 7–13): 21–34 (8–18 + 12–17): 24–38 (12–19 + 12–19): 25–36 (12–17 + 12–19): 23–34 (12–17 + 11–18): 23–33 (11–17 + 11–16): 19–30 (8–15 + 9–15), sternite XI with 8–12 (4–6 + 4–6) and with a pair of long tactile setae. Anterior genital operculum with 27–41 setae and posterior operculum with 11–19 setae.

Females (29 specimens analysed) (Table S2). Chaetotaxy of carapace: 73–99 setae, 35–49 of them situated in front of anterior transverse furrow, 23–36 on medial disk, posterior margin with 10–17 setae. Cheliceral galea with 5–6 short terminal rami, serrula exterior with 17 blades. Pedipalpal coxa excluding manducatory process with 21–24 setae, manducatory process with 3

setae and 1 microseta; coxal chaetotaxy of legs I–IV: 16–20: 18–23: 20–23: 32–34. Lyrifissures: none or one on pedipalpal coxa, one on each pedal coxa I–IV; each pedipalpal coxa with two maxillary lyrifissures. Palps: fixed chelal finger with 30–35 and movable chelal finger with 31–37 marginal teeth; fixed chelal finger with 4–7 antiaxial accessory teeth and movable chelal finger with 4–5 antiaxial accessory teeth. Chaetotaxy of tergites I–X: 15–21 (left hemitergite 7–11 + right hemitergite 7–11): 15–21 (8–11 + 6–11): 14–18 (7–9 + 6–10): 16–25 (8–13 + 8–12): 18–27 (9–13 + 9–14): 20–28 (10–14 + 10–14): 19–28 (9–14 + 10–15): 20–27 (10–14 + 9–14): 10–28 (9–15 + 10–13): 18–24 (9–13 + 9–12); tergite XI with 10–12 setae (5–6 + 5–6) plus a pair of long tactile setae. Chaetotaxy of sternites IV–X: 6–13 (left hemisternite 3–6 + right hemisternite 3–7): 22–33 (10–17 + 11–17): 26–36 (12–18 + 13–19): 27–36 (13–20 + 12–18): 26–37 (13–19 + 11–19): 24–33 (13–17 + 11–16): 19–27 (9–14 + 10–14); sternite XI with 10–12 setae (5–6 + 5–6) plus a pair of long tactile setae. Anterior genital operculum with 21–29 setae and posterior operculum with 9–15 setae.

Lamprochernes nodosus

Material examined:
Other material: For details see Tables S1 and S9.

Males (8 specimens analysed) (Table S2): Chaetotaxy of carapace: 85–110 setae, 38–50 of them on anterior disk, 24–38 on medial disk, posterior margin with 15–22 setae. Cheliceral galea with 5 short terminal rami, serrula exterior with 17–18 blades. Pedipalpal coxa excluding manducatory process with 19–22 setae, manducatory process with 3 setae and 1 microseta; coxal chaetotaxy of legs I–IV: 13–14: 15–16: 15–19: 24–29. Lyrifissures: one on pedipalpal coxa, one on each pedal coxa I and IV and one or two on pedal coxa II and III; each pedipalpal coxa with two maxillary lyrifissures. Palps: fixed chelal finger with 30–34 and movable chelal finger with 33–36 marginal teeth; both fixed and movable chelal finger with 3–5 antiaxial accessory teeth. Chaetotaxy of tergites I–XI: 20–22 (left hemitergite 9–11 + right hemitergite 10–12): 20–24 (10–12 + 9–13): 18–20 (9–10 + 8–10): 22–25 (10–13 + 11–13): 22–27 (11–14 + 11–14): 23–28 (11–14 + 12–14): 22–27 (11–14 + 11–14): 23–29 (12–14 + 11–15): 22–27 (10–14 + 10–13): 17–21 (9–11 + 8–10), tergite XI with 8–10 setae (4–5 + 4–5) and with a pair of long tactile setae. Chaetotaxy of sternites IV–XI: 12–14 (left hemisternite 6–8 + right hemisternite 5–8): 26–31 (13–16 + 12–15): 27–31 (13–16 + 14–15): 29–33 (15–17 + 14–17): 28–36 (14–17 + 13–19): 26–31 (13–15 + 12–16): 17–25 (9–12 + 8–13), sternite XI with 8–10

(4–5 + 4–5) and with a pair of long tactile setae. Anterior genital operculum with 18–23 setae and posterior operculum with 9–12 setae.

Females (13 specimens analysed) (Table S2). Chaetotaxy of carapace: 81–101 setae, 33–47 of them situated on anterior disk, 27–38 on medial disk, posterior margin with 17–21 setae. Cheliceral galea with 5–6 short terminal rami, serrula exterior with 17–18 blades. Pedipalpal coxa excluding manducatory process with 14–19 setae, manducatory process with 3 setae and 1 microseta; coxal chaetotaxy of legs I–IV: 12–15: 14–19: 16–20: 31–35. Lyrifissures: one on pedipalpal coxa, one on each pedal coxa I and IV, 1–2 on each pedal coxa III and IV; each pedipalpal coxa with two maxillary lyrifissures. Palps: fixed chelal finger with 30–35 and movable chelal finger with 31–38 marginal teeth; both fixed and movable chelal finger with 3–5 antiaxial accessory teeth. Chaetotaxy of tergites I–XI: 19–23 (left hemitergite 9–12 + right hemitergite 9–12): 20–26 (10–13 + 9–13): 16–22 (8–11 + 8–11): 23–29 (10–15+ 11–14): 24–32 (11–16 + 12–16): 23–33 (11–17 + 12–16): 24–33 (11–17 + 13–16): 23–30 (12–15 + 11–15): 22–28 (11–15 + 11–14): 17–22 (8–12 + 8–12), tergite XI with 8–10 setae (4–5 + 4–5) and with a pair of long tactile setae. Chaetotaxy of sternites IV–XI: 6–9 (left hemisternite 3–5 + right hemisternite 3–5): 23–33 (11–17 + 12–16): 26–36 (13–19 + 13–19): 30–40 (15–19 + 14–21): 28–34 (14–18 + 14–18): 27–37 (12–18 + 13–19): 20–24 (9–12 + 9–12), sternite XI with 8–10 (4–5 + 4–5) and with a pair of long tactile setae. Anterior genital operculum with 17–22 setae and posterior operculum with 6–9 setae.
