# Supplementary material for: Integrative Taxonomy Approach Reveals Cryptic Diversity within the Phoretic Pseudoscorpion Genus Lamprochernes (Pseudoscorpiones: Chernetidae)
Source: Insects. 2023 Jan 25;14(2):122. doi: 10.3390/insects14020122 (PMC9964657; doi:10.3390/insects14020122)
Supplement: Supplementary file 1 [file insects-14-00122-s001.zip › supplementary tables/Table S2.pdf]

Table S2. Descriptive statistics of measured morphological characters of the studied *Lamprochernes* species. Abbreviation: n – number of measured specimens. Mean values of the measured characters ± standard deviation (Mean±SD) are given in upper rows; minimum and maximum (Min–Max) are in lower rows. Values of all the measured characters are in mm.

**Characters/ Species**	***Lamprochernes abditus* sp. nov.**		***Lamprochernes chyzeri***		***Lamprochernes nodosus***	
**Mean±SD**	Males	Females	Males	Females	Males	Females
**Min–Max**	n = 4	n = 7	n = 66	n = 29	n = 8	n = 13
Body length	2.29±0.23	2.67±0.34	2.25±0.20	2.58±0.38	1.94±0.17	2.45±0.42
	1.95–2.44	2.29–3.08	1.71–2.56	1.94–3.13	1.75–2.27	1.83–3.24
Carapace length	0.69±0.03	0.72±0.03	0.67±0.03	0.70±0.03	0.57±0.02	0.62±0.04
	0.64–0.71	0.67–0.76	0.60–0.72	0.61–0.77	0.54–0.61	0.56–0.69
Carapace posterior width	0.56±0.05	0.57±0.03	0.50±0.04	0.55±0.05	0.44±0.03	0.50±0.03
	0.51–0.63	0.53–0.61	0.43–0.58	0.42–0.64	0.40–0.48	0.46–0.56
Carapace length/posterior width ratio	1.24±0.08	1.25±0.07	1.35±0.08	1.29±0.09	1.31±0.05	1.23±0.05
	1.13–1.33	1.15–1.36	1.16–1.51	1.13–1.55	1.25–1.40	1.17–1.35
Chelicera length	0.18±0.01	0.18±0.01	0.18±0.01	0.19±0.01	0.18±0.01	0.19±0.01
	0.17–0.19	0.16–0.20	0.16–0.21	0.17–0.21	0.17–0.18	0.17–0.21
Chelicera width	0.10±0.01	0.10±0.01	0.10±0.01	0.10±0.01	0.10±0.01	0.10±0.01
	0.09–0.11	0.09–0.11	0.08–0.11	0.09–0.12	0.09–0.10	0.09–0.11

**Characters/ Species**	***Lamprochernes abditus* sp. nov.**		***Lamprochernes chyzeri***		***Lamprochernes nodosus***	
**Mean±SD**	Males	Females	Males	Females	Males	Females
**Min–Max**	n = 4	n = 7	n = 66	n = 29	n = 8	n = 13
Chelicera length/width ratio	1.83±0.20	1.87±0.11	1.83±0.16	1.79±0.20	1.82±0.13	1.86±0.18
	1.55–2.00	1.73–2.00	1.55–2.38	1.50–2.22	1.70–2.00	1.64–2.22
Cheliceral movable finger length	0.17±0.00	0.17±0.00	0.16±0.01	0.17±0.01	0.16±0.01	0.17±0.01
	0.17–0.17	0.17–0.17	0.15–0.18	0.15–0.18	0.15–0.17	0.16–0.18
Palpal trochanter length	0.35±0.01	0.34±0.02	0.34±0.02	0.35±0.02	0.28±0.02	0.30±0.02
	0.35–0.36	0.33–0.37	0.27–0.38	0.30–0.39	0.26–0.31	0.27–0.32
Palpal trochanter width	0.23±0.01	0.22±0.02	0.21±0.02	0.21±0.02	0.16±0.01	0.17±0.01
	0.22–0.23	0.20–0.26	0.17–0.24	0.17–0.26	0.14–0.17	0.15–0.19
Palpal trochanter length/width ratio	1.55±0.06	1.58±0.11	1.65±0.10	1.65±0.14	1.76±0.06	1.80±0.12
	1.52–1.64	1.35–1.68	1.36–1.88	1.35–1.88	1.69–1.86	1.53–2.00
Palpal femur length	0.57±0.01	0.56±0.02	0.53±0.03	0.54±0.04	0.41±0.03	0.44±0.03
	0.55–0.57	0.54–0.59	0.44–0.61	0.46–0.62	0.36–0.43	0.40–0.49
Palpal femur width	0.24±0.01	0.24±0.01	0.22±0.02	0.23±0.02	0.19±0.01	0.20±0.01
	0.24–0.25	0.23–0.26	0.18–0.27	0.19–0.26	0.18–0.20	0.18–0.22
Palpal femur length/width ratio	2.33±0.09	2.31±0.08	2.37±0.10	2.38±0.08	2.16±0.08	2.20±0.10
	2.20–2.38	2.23–2.46	2.16–2.68	2.19–2.57	2.00–2.26	2.00–2.33

**Characters/ Species**	***Lamprochernes abditus* sp. nov.**		***Lamprochernes chyzeri***		***Lamprochernes nodosus***	
**Mean±SD**	Males	Females	Males	Females	Males	Females
**Min–Max**	n = 4	n = 7	n = 66	n = 29	n = 8	n = 13
Palpal patella length	0.56±0.01	0.56±0.02	0.53±0.03	0.54±0.04	0.41±0.03	0.44±0.03
	0.55–0.56	0.52–0.58	0.45–0.62	0.47–0.62	0.36–0.44	0.41–0.49
Palpal patella width	0.25±0.01	0.25±0.01	0.24±0.02	0.25±0.02	0.20±0.02	0.22±0.01
	0.24–0.26	0.24–0.27	0.20–0.27	0.21–0.28	0.18–0.22	0.20–0.24
Palpal patella length/width ratio	2.20±0.06	2.19±0.06	2.21±0.09	2.17±0.07	2.05±0.10	2.04±0.06
	2.15–2.29	2.12–2.29	2.00–2.50	2.04–2.30	1.86–2.20	1.91–2.15
Palpal hand with pedicel length	0.52±0.00	0.55±0.01	0.52±0.03	0.54±0.04	0.41±0.01	0.47±0.03
	0.52–0.52	0.54–0.58	0.44–0.60	0.46–0.61	0.40–0.42	0.43–0.51
Palpal hand without pedicel length	0.45±0.01	0.48±0.01	0.45±0.03	0.47±0.03	0.36±0.01	0.41±0.02
	0.45–0.46	0.47–0.50	0.38–0.53	0.39–0.53	0.33–0.37	0.38–0.46
Palpal hand width	0.29±0.01	0.30±0.01	0.28±0.02	0.31±0.02	0.26±0.02	0.30±0.02
	0.27–0.30	0.29–0.33	0.24–0.32	0.24–0.35	0.23–0.27	0.27–0.33
Palpal hand with pedicel length/width ratio	1.83±0.08	1.84±0.08	1.85±0.08	1.77±0.07	1.62±0.09	1.57±0.09
	1.73–1.93	1.67–1.93	1.68–2.07	1.68–1.92	1.56–1.83	1.45–1.74
Palpal movable finger length	0.46±0.01	0.47±0.02	0.44±0.02	0.45±0.03	0.39±0.02	0.41±0.02
	0.45–0.47	0.44–0.49	0.37–0.49	0.37–0.51	0.36–0.41	0.37–0.44

**Characters/ Species**	*Lamprochernes abditus* **sp. nov.**		*Lamprochernes chyzeri*		*Lamprochernes nodosus*	
**Mean±SD**	Males	Females	Males	Females	Males	Females
**Min–Max**	n = 4	n = 7	n = 66	n = 29	n = 8	n = 13
Palpal chela length	0.94±0.01	0.97±0.03	0.91±0.04	0.94±0.06	0.76±0.03	0.83±0.04
	0.92–0.95	0.94–1.02	0.77–1.01	0.79–1.04	0.71–0.79	0.78–0.91
Palpal chela length/hand width ratio	3.29±0.18	3.24±0.11	3.26±0.11	3.07±0.13	2.96±0.12	2.75±0.11
	3.10–3.48	3.03–3.40	2.97–3.52	2.82–3.31	2.88–3.22	2.58–2.93
Leg I trochanter length	0.12±0.01	0.12±0.01	0.12±0.01	0.13±0.01	0.11±0.01	0.12±0.01
	0.11–0.12	0.11–0.13	0.10–0.15	0.11–0.14	0.10–0.12	0.11–0.13
Leg I trochanter width	0.10±0.00	0.10±0.00	0.10±0.01	0.10±0.01	0.09±0.01	0.10±0.01
	0.10–0.10	0.09–0.10	0.09–0.11	0.09–0.12	0.09–0.10	0.09–0.11
Leg I trochanter length/width ratio	1.15±0.06	1.22±0.07	1.25±0.09	1.22±0.08	1.18±0.05	1.18±0.08
	1.10–1.20	1.10–1.30	1.09–1.56	1.09–1.40	1.10–1.22	1.09–1.33
Leg I femur length	0.15±0.01	0.16±0.01	0.15±0.01	0.15±0.01	0.12±0.01	0.14±0.01
	0.14–0.16	0.15–0.16	0.12–0.18	0.13–0.17	0.11–0.14	0.12–0.15
Leg I femur width	0.11±0.00	0.12±0.01	0.11±0.01	0.11±0.01	0.10±0.01	0.11±0.01
	0.11–0.11	0.11–0.12	0.10–0.12	0.10–0.13	0.08–0.11	0.10–0.12
Leg I femur length/width ratio	1.36±0.07	1.35±0.06	1.36±0.10	1.34±0.09	1.28±0.16	1.28±0.07
	1.27–1.45	1.25–1.45	1.17–1.60	1.18–1.50	1.09–1.50	1.18–1.40

Characters/ Species	*Lamprochernes abditus* **sp. nov.**		*Lamprochernes chyzeri*		*Lamprochernes nodosus*	
**Mean±SD**	Males	Females	Males	Females	Males	Females
**Min–Max**	n = 4	n = 7	n = 66	n = 29	n = 8	n = 13
Leg I patella length	0.28±0.02	0.28±0.01	0.27±0.02	0.28±0.02	0.22±0.02	0.23±0.01
	0.26–0.29	0.27–0.29	0.20–0.30	0.24–0.31	0.18–0.23	0.21–0.25
Leg I patella width	0.12±0.01	0.11±0.01	0.11±0.01	0.11±0.01	0.10±0.01	0.10±0.01
	0.11–0.12	0.10–0.12	0.09–0.12	0.09–0.13	0.09–0.10	0.09–0.11
Leg I patella length/width ratio	2.41±0.19	2.50±0.17	2.49±0.14	2.52±0.14	2.28±0.15	2.41±0.14
	2.17–2.64	2.33–2.80	2.18–2.89	2.27–2.82	2.00–2.56	2.09–2.67
Leg I tibia length	0.25±0.00	0.26±0.01	0.25±0.02	0.26±0.02	0.20±0.01	0.23±0.01
	0.25–0.25	0.25–0.27	0.22–0.29	0.22–0.29	0.18–0.22	0.21–0.25
Leg I tibia width	0.08±0.01	0.08±0.00	0.07±0.01	0.08±0.01	0.06±0.01	0.07±0.00
	0.07–0.08	0.08–0.09	0.07–0.08	0.06–0.09	0.06–0.07	0.07–0.08
Leg I tibia length/width ratio	3.24±0.22	3.23±0.13	3.38±0.20	3.46±0.22	3.13±0.33	3.19±0.19
	3.13–3.57	3.00–3.38	2.88–3.86	2.88–3.86	2.86–3.67	3.00–3.57
Leg I tarsus length	0.22±0.01	0.22±0.01	0.23±0.01	0.24±0.01	0.21±0.02	0.23±0.01
	0.21–0.23	0.21–0.24	0.20–0.25	0.20–0.26	0.19–0.24	0.21–0.25
Leg I tarsus width	0.05±0.00	0.06±0.00	0.05±0.00	0.05±0.01	0.05±0.01	0.05±0.00
	0.05–0.05	0.05–0.06	0.05–0.06	0.05–0.06	0.04–0.05	0.05–0.06

Characters/ Species	*Lamprochernes abditus* **sp. nov.**		*Lamprochernes chyzeri*		*Lamprochernes nodosus*	
**Mean±SD**	Males	Females	Males	Females	Males	Females
**Min–Max**	n = 4	n = 7	n = 66	n = 29	n = 8	n = 13
Leg I tarsus length/width ratio	4.40±0.16	3.92±0.38	4.30±0.30	4.32±0.31	4.64±0.39	4.60±0.28
	4.20–4.60	3.50–4.60	3.33–4.80	3.67–4.80	4.20–5.25	4.17–5.00
Leg IV trochanter length	0.19±0.01	0.20±0.02	0.19±0.01	0.20±0.01	0.18±0.01	0.20±0.02
	0.18–0.20	0.18–0.23	0.16–0.22	0.19–0.23	0.17–0.19	0.18–0.23
Leg IV trochanter width	0.12±0.01	0.12±0.01	0.11±0.01	0.12±0.01	0.11±0.01	0.11±0.01
	0.11–0.12	0.11–0.13	0.10–0.13	0.10–0.14	0.10–0.11	0.10–0.12
Leg IV trochanter length/width ratio	1.65±0.06	1.70±0.08	1.72±0.13	1.74±0.10	1.69±0.07	1.83±0.18
	1.58–1.73	1.58–1.82	1.38–2.00	1.54–1.90	1.64–1.80	1.64–2.10
Leg IV femoropatella length	0.49±0.02	0.51±0.02	0.48±0.03	0.51±0.04	0.42±0.03	0.47±0.04
	0.47–0.51	0.48–0.55	0.40–0.55	0.44–0.58	0.38–0.44	0.41–0.56
Leg IV femoropatella width	0.15±0.01	0.15±0.01	0.15±0.01	0.15±0.01	0.12±0.01	0.12±0.01
	0.14–0.16	0.14–0.16	0.13–0.17	0.13–0.17	0.11–0.13	0.11–0.14
Leg IV femoropatella length/width ratio	3.20±0.15	3.37±0.17	3.22±0.17	3.51±0.26	3.35±0.11	3.81±0.27
	3.00–3.36	3.19–3.67	2.88–3.57	2.94–4.08	3.17–3.55	3.29–4.25
Leg IV tibia length	0.38±0.01	0.38±0.02	0.37±0.02	0.38±0.03	0.29±0.01	0.32±0.02
	0.37–0.39	0.36–0.40	0.31–0.42	0.32–0.42	0.28–0.31	0.30–0.37

**Characters/ Species**	***Lamprochernes abditus* sp. nov.**		***Lamprochernes chyzeri***		***Lamprochernes nodosus***	
**Mean±SD**	Males	Females	Males	Females	Males	Females
**Min–Max**	n = 4	n = 7	n = 66	n = 29	n = 8	n = 13
Leg IV tibia width	0.10±0.01	0.10±0.00	0.10±0.01	0.10±0.01	0.08±0.00	0.09±0.01
	0.10–0.11	0.10–0.11	0.08–0.11	0.08–0.11	0.08–0.09	0.08–0.10
Leg IV tibia length/width ratio	3.67±0.22	3.75±0.13	3.70±0.18	3.88±0.21	3.60±0.16	3.80±0.22
	3.36–3.90	3.60–4.00	3.18–4.11	3.55–4.33	3.44–3.88	3.20–4.11
Leg IV tarsus length	0.27±0.01	0.27±0.02	0.28±0.01	0.29±0.02	0.27±0.01	0.28±0.01
	0.26–0.28	0.25–0.30	0.24–0.32	0.26–0.32	0.25–0.28	0.26–0.32
Leg IV tarsus width	0.07±0.01	0.07±0.01	0.06±0.00	0.07±0.01	0.06±0.00	0.06±0.00
	0.06–0.07	0.06–0.07	0.05–0.07	0.06–0.07	0.06–0.06	0.06–0.07
Leg IV tarsus length/width ratio	4.01±0.22	4.16±0.23	4.52±0.26	4.45±0.33	4.45±0.19	4.66±0.17
	3.86–4.33	3.86–4.50	3.86–4.83	3.71–5.17	4.17–4.67	4.33–5.00
