# Supplementary material for: Integrative Taxonomy Approach Reveals Cryptic Diversity within the Phoretic Pseudoscorpion Genus Lamprochernes (Pseudoscorpiones: Chernetidae)
Source: Insects. 2023 Jan 25;14(2):122. doi: 10.3390/insects14020122 (PMC9964657; doi:10.3390/insects14020122)
Supplement: Supplementary file 1 [file insects-14-00122-s001.zip › supplementary tables/Table S4.pdf]

Table S4. Uncorrected p-distances, Tamura – Nei genetic distances within *Lamprochernes* species. Abbreviations: H – number of haplotypes, S.E. – standard error.

**Species**	**H**	**p-distance [S.E.]**	**Tamura – Nei [S.E.]**
Lamprochernes abditus sp. nov.	5	0.0095 [0.0027]	0.0096 [0.0027]
Lamprochernes chyzeri	24	0.0066 [0.0015]	0.0067 [0.0015]
Lamprochernes nodosus	10	0.0094 [0.0024]	0.0095 [0.0024]
Lamprochernes savignyi	6	0.0095 [0.0027]	0.0096 [0.0026]
