# Supplementary material for: Integrative Taxonomy Approach Reveals Cryptic Diversity within the Phoretic Pseudoscorpion Genus Lamprochernes (Pseudoscorpiones: Chernetidae)
Source: Insects. 2023 Jan 25;14(2):122. doi: 10.3390/insects14020122 (PMC9964657; doi:10.3390/insects14020122)
Supplement: Supplementary file 1 [file insects-14-00122-s001.zip › supplementary tables/Table S5.pdf]

Table S5. Uncorrected p-distances, Tamura – Nei genetic distances among *Lamprochernes* species. Abbreviation: S.E. – Standard error.

**Species**	***Lamprochernes abditus* sp. nov. p-distance [S.E.], Tamura – Nei [S.E.]**	***Lamprochernes chyzeri* p-distance [S.E.], Tamura – Nei [S.E.]**	***Lamprochernes nodosus* p-distance [S.E.], Tamura – Nei [S.E.]**
L. abditus sp. nov.			
L. chyzeri	0.1109 [0.0121], 0.1220 [0.0149]		
L. nodosus	0.1779 [0.0149], 0.2082 [0.0205]	0.1711 [0.0148], 0.1979 [0.0205]	
L. savignyi	0.1757 [0.0152], 0.2035 [0.0203]	0.1692 [0.0149], 0.1959 [0.0206]	0.1728 [0.0148], 0.2001 [0.0205]
