# Supplementary material for: Integrative Taxonomy Approach Reveals Cryptic Diversity within the Phoretic Pseudoscorpion Genus Lamprochernes (Pseudoscorpiones: Chernetidae)
Source: Insects. 2023 Jan 25;14(2):122. doi: 10.3390/insects14020122 (PMC9964657; doi:10.3390/insects14020122)
Supplement: Supplementary file 1 [file insects-14-00122-s001.zip › supplementary tables/Table S6.pdf]

Table S6. Measurements of the relative diploid set length (%DSL) and arm ratio (AR) of chromosomes including standard deviation (±SD) of *Lamprochernes* species.

**Species/ Chromosome pair No.**	***L. abditus* sp. nov. 2n male = 63**		***L. chyzeri* 2n male = 63**		***L. nodosus* 2n female = 64**		***L. savignyi* 2n male = 41**	
	**%DSL (±SD)**	**AR (±SD)**	**%DSL (±SD)**	**AR (±SD)**	**%DSL (±SD)**	**AR (±SD)**	**%DSL (±SD)**	**AR (±SD)**
1	2.69 (±0.29)	2.12 (±0.35)	2.67 (±0.28)	1.87 (±0.49)	3.01 (±0.31)	1.33 (±0.25)	3.30 (±0.12)	1.35 (±0.18)
2	2.62 (±0.21)	1.35 (±0.17)	2.51 (±0.22)	1.30 (±0.20)	2.47 (±0.24)	1.36 (±0.18)	3.09 (±0.11)	1.21 (±0.09)
3	2.35 (±0.16)	1.33 (±0.20)	2.22 (±0.33)	2.28 (±0.46)	2.27 (±0.18)	2.90 (±0.66)	2.99 (±0.08)	1.31 (±0.23)
4	2.32 (±0.13)	1.49 (±0.16)	2.13 (±0.14)	1.17 (±0.12)	2.15 (±0.08)	2.16 (±0.54)	2.90 (±0.08)	1.26 (±0.19)
5	2.24 (±0.15)	2.31 (±0.41)	2.12 (±0.16)	2.27 (±0.84)	2.05 (±0.28)	1.16 (±0.10)	2.79 (±0.06)	3.59 (±0.52)
6	2.16 (±0.12)	1.65 (±0.30)	2.02 (±0.13)	1.22 (±0.18)	1.94 (±0.10)	1.42 (±0.20)	2.78 (±0.50)	1.25 (±0.15)
7	2.04 (±0.11)	3.14 (±0.72)	1.95 (±0.13)	2.36 (±0.39)	1.83 (±0.11)	1.31 (±0.18)	2.77 (±0.09)	1.38 (±0.20)
8	2.04 (±0.14)	1.29 (±0.16)	1.84 (±0.49)	1.63 (±0.42)	1.80 (±0.13)	2.45 (±0.52)	2.72 (±0.04)	1.26 (±0.18)
9	1.89 (±0.11)	1.34 (±0.18)	1.81 (±0.21)	1.49 (±0.30)	1.75 (±0.05)	1.36 (±0.19)	2.60 (±0.06)	1.94 (±0.42)
10	1.86 (±0.17)	1.27 (±0.16)	1.80 (±0.17)	1.18 (±0.15)	1.67 (±0.09)	1.49 (±0.31)	2.52 (±0.07)	1.29 (±0.18)
11	1.86 (±0.18)	2.16 (±0.38)	1.77 (±0.23)	5.36 (±1.88)	1.66 (±0.10)	3.22 (±0.75)	2.43 (±0.07)	1.28 (±0.20)
12	1.75 (±0.16)	1.43 (±0.24)	1.71 (±0.13)	2.39 (±1.27)	1.58 (±0.06)	1.43 (±0.34)	2.35 (±0.07)	1.57 (±0.76)
13	1.73 (±0.09)	1.98 (±0.17)	1.63 (±0.13)	1.47 (±0.45)	1.51 (±0.07)	1.92 (±0.36)	2.33 (±0.14)	3.16 (±0.97)
14	1.55 (±0.12)	1.51 (±0.24)	1.58 (±0.15)	5.16 (±1.95)	1.50 (±0.06)	1.24 (±0.24)	2.13 (±0.16)	3.42 (±0.74)
15	1.52 (±0.09)	2.57 (±0.48)	1.52 (±0.17)	≥7.00	1.47 (±0.07)	2.65 (±0.73)	2.10 (±0.10)	1.41 (±0.24)

Species/ Chromosome pair No.	*L. abditus* sp. nov. 2n male = 63		*L. chyzeri* 2n male = 63		*L. nodosus* 2n female = 64		*L. savignyi* 2n male = 41	
	%DSL (±SD)	AR (±SD)	%DSL (±SD)	AR (±SD)	%DSL (±SD)	AR (±SD)	%DSL (±SD)	AR (±SD)
16	1.44 (±0.11)	1.18 (±0.13)	1.50 (±0.14)	1.29 (±0.14)	1.40 (±0.12)	1.26 (±0.19)	2.02 (±0.17)	1.92 (±0.27)
17	1.40 (±0.14)	1.92 (±0.09)	1.48 (±0.13)	1.27 (±0.28)	1.39 (±0.08)	3.61 (±2.03)	1.92 (±0.12)	1.36 (±0.25)
18	1.39 (±0.07)	1.37 (±0.17)	1.42 (±0.17)	≥7.00	1.37 (±0.08)	1.52 (±0.42)	1.74 (±0.12)	3.03 (±1.02)
19	1.30 (±0.13)	1.45 (±0.24)	1.42 (±0.14)	1.25 (±0.17)	1.34 (±0.07)	2.34 (±1.01)	1.38 (±0.22)	2.21 (±0.26)
20	1.26 (±0.07)	2.17 (±0.27)	1.40 (±0.07)	1.41 (±0.33)	1.28 (±0.07)	4.17 (±1.35)	1.32 (±0.27)	1.29 (±0.22)
21	1.19 (±0.14)	3.19 (±0.66)	1.36 (±0.14)	2.32 (±0.64)	1.23 (±0.04)	2.03 (±0.33)	–	–
22	1.18 (±0.15)	1.28 (±0.21)	1.23 (±0.14)	1.42 (±0.27)	1.17 (±0.08)	≥7.00	–	–
23	1.17 (±0.09)	1.91 (±0.25)	1.23 (±0.18)	≥7.00	1.17 (±0.03)	1.56 (±0.30)	–	–
24	1.09 (±0.11)	1.37 (±0.18)	1.19 (±0.07)	3.38 (±2.11)	1.14 (±0.08)	3.36 (±0.46)	–	–
25	1.02 (±0.07)	1.41 (±0.26)	1.13 (±0.09)	≥7.00	1.13 (±0.04)	2.07 (±0.54)	–	–
26	0.99 (±0.10)	5.35 (±3.49)	1.13 (±0.11)	5.12 (±1.64)	1.10 (±0.07)	1.26 (±0.24)	–	–
27	0.94 (±0.09)	2.04 (±0.35)	1.11 (±0.11)	1.35 (±0.18)	1.01 (±0.06)	3.27 (±0.92)	–	–
28	0.89 (±0.83)	≥7.00	1.07 (±0.10)	4.51 (±0.93)	1.00 (±0.07)	1.32 (±0.28)	–	–
29	0.88 (±0.14)	2.48 (±0.40)	0.98 (±0.11)	≥7.00	0.94 (±0.05)	≥7.00	–	–
30	0.85 (±0.06)	1.28 (±0.17)	0.94 (±0.10)	≥7.00	0.93 (±0.06)	2.45 (±0.50)	–	–
31	0.82 (±0.09)	≥7.00	0.87 (±0.12)	2.24 (±0.64)	0.85 (±0.12)	4.18 (±1.84)	–	–
X	3.14 (±0.46)	2.37 (±1.07)	3.15 (±0.24)	2.13 (±0.29)	2.89 (±0.35)	1.83 (±0.14)	3.69 (±0.68)	1.47 (±0.28)
