# Supplementary material for: Integrative Taxonomy Approach Reveals Cryptic Diversity within the Phoretic Pseudoscorpion Genus Lamprochernes (Pseudoscorpiones: Chernetidae)
Source: Insects. 2023 Jan 25;14(2):122. doi: 10.3390/insects14020122 (PMC9964657; doi:10.3390/insects14020122)
Supplement: Supplementary file 1 [file insects-14-00122-s001.zip › supplementary tables/Table S7.pdf]

Table S7. The list of the analyzed characters and the results of the canonical discriminant analyses CDA 1 and CDA 2 (see Figure S3). The total canonical structure values that express the correlations of the morphological characters with the canonical axes (Can 1 to Can 2) are listed with the highest values shown in bold. The acronym NA (not applicable) indicates that the character was not included in the respective analysis because it was removed from the dataset based on the results of the preceding stepwise discriminant analysis.

**Characters**	**CDA1**		**CDA2**	
	**Can 1**	**Can 2**	**Can 1**	**Can 2**
Carapace total setae number	-0.490	-0.069	NA	NA
Carapace setae number in front of anterior transverse furrow	-0.359	**-0.218**	NA	NA
Carapace setae number on medial disk	NA	NA	NA	NA
Carapace setae number behind posterior transverse furrow	**-0.616**	**0.471**	**-0.866**	-0.153
Number of marginal teeth fixed finger	NA	NA	NA	NA
Number of antiaxial accessory teeth fixed finger	NA	NA	NA	NA
Number of marginal teeth movable finger	-0.050	**0.202**	NA	NA
Number of antiaxial accessory teeth movable finger	NA	NA	NA	NA
Setae number on tergite I	NA	NA	NA	NA
Setae number on tergite II	**-0.660**	0.017	NA	NA
Setae number on tergite III	**-0.628**	0.152	NA	NA
Setae number on tergite IV	NA	NA	NA	NA
Setae number on tergite V	NA	NA	NA	NA
Setae number on tergite VI	-0.468	**-0.210**	NA	NA
Setae number on tergite VII	NA	NA	NA	NA
Setae number on tergite VIII	-0.414	-0.165	**-0.657**	0.176
Setae number on tergite IX	NA	NA	NA	NA
Setae number on tergite X	NA	NA	NA	NA
Setae number on sternite IV	NA	NA	NA	NA
Setae number on sternite V	NA	NA	0.503	**0.325**
Setae number on sternite VI	NA	NA	NA	NA
Setae number on sternite VII	-0.301	**-0.283**	NA	NA
Setae number on sternite VIII	NA	NA	NA	NA
Setae number on sternite IX	NA	NA	NA	NA
Setae number on sternite X	0.233	**-0.227**	NA	NA
Setae number on anterior genital operculum	**0.670**	-0.096	NA	NA
Setae number on posterior genital operculum	**0.577**	-0.024	**0.793**	0.176
Palpal trochanter length	0.510	0.069	NA	NA
Palpal trochanter width	NA	NA	NA	NA
Palpal femur length	**0.704**	0.113	**0.899**	-0.029
Palpal femur width	NA	NA	NA	NA
Palpal patella length	NA	NA	NA	NA
Palpal patella width	NA	NA	NA	NA
Palpal hand with pedicel length	NA	NA	NA	NA
Palpal hand without pedicel length	NA	NA	NA	NA
Palpal hand width	0.322	0.062	0.216	0.152
Palpal movable finger length	NA	NA	**0.761**	-0.096
Palpal chela length	NA	NA	NA	NA
Leg I trochanter length	0.168	**-0.270**	NA	NA
Leg I trochanter width	NA	NA	NA	NA
Leg I femur length	0.510	0.027	NA	NA
Leg I femur width	NA	NA	NA	NA
Leg I patella length	NA	NA	NA	NA
Leg I patella width	NA	NA	NA	NA
Leg I tibia length	NA	NA	NA	NA
Leg I tibia width	NA	NA	NA	NA
Leg I tarsus length	NA	NA	NA	NA
Leg I tarsus width	NA	NA	NA	NA
Leg IV trochanter length	NA	NA	NA	NA
Leg IV trochanter width	NA	NA	NA	NA
Leg IV femoropatella length	0.438	0.029	NA	NA
Leg IV femoropatella width	NA	NA	**0.772**	-0.056
Leg IV tibia length	**0.662**	-0.021	NA	NA
Leg IV tibia width	0.064	-0.040	NA	NA
Leg IV tarsus length	0.107	**-0.237**	0.284	**0.547**
Leg IV tarsus width	NA	NA	NA	NA
