# Supplementary material for: Integrative Taxonomy Approach Reveals Cryptic Diversity within the Phoretic Pseudoscorpion Genus Lamprochernes (Pseudoscorpiones: Chernetidae)
Source: Insects. 2023 Jan 25;14(2):122. doi: 10.3390/insects14020122 (PMC9964657; doi:10.3390/insects14020122)
Supplement: Supplementary file 1 [file insects-14-00122-s001.zip › supplementary tables/Table S8.pdf]

Table S8. Classification functions for males and females of *Lamprochernes abditus* sp. nov., *L. chyzeri* and *L. nodosus* derived from linear classificatory discriminant analyses (LDA 1 and LDA 2 respectively). They can be used to estimate the taxonomic identity of an unknown individual. When values of characters of the unknown individual are inserted into each formula, the unknown individual is classified into the taxon that shows the highest score.

Sex	Species	Classification function
Males (LDA 1)	*L. abditus* sp. nov.	f(x)= -787.414 +3.325*Total setae number -1.379*Setae car anterior -3.439*Setae car posterior +25.304*Marginal teeth movable finger +8.656*Tergite II +6.165*Tergite III +7.902*Tergite VI +0.103*Tergite VIII +7.332*Sternite VII -6.533*Sternite X -0.865*Go anterior +2.822*Go posterior +841.200*Palp troch length -767.690*Palpal femur length +2117.979*Palpal hand width -1704.198*Leg I troch length +336.352*Leg I femur length -324.307*Leg IV femoropatella length -425.431*Leg IV tibia length +55.445*Leg IV tibia width +125.829*Leg IV tarsus length
	L. chyzeri	f(x)= -829.857 +3.763*Total setae number -0.123*Setae car anterior -8.410*Setae car posterior +25.533*Marginal teeth movable finger +9.447*Tergite II +3.127*Tergite III +10.099*Tergite VI +2.773*Tergite VIII +6.350*Sternite VII -7.624*Sternite X -0.577*Go anterior +1.309*Go posterior +1089.962*Palp troch length -1424.975*Palpal femur length +2148.837*Palpal hand width -868.036*Leg I troch length -48.334*Leg I femur length -117.989*Leg IV femoropatella length -566.884*Leg IV tibia length +90.928*Leg IV tibia width +809.267*Leg IV tarsus length
	L. nodosus	f(x)= -1302.044 +1.072*Total setae number +5.185*Setae car anterior -3.508*Setae car posterior +33.670*Marginal teeth movable finger +20.406*Tergite II +16.538*Tergite III +8.078*Tergite VI +7.409*Tergite VIII +11.507*Sternite VII -16.694*Sternite X -3.565*Go anterior -9.216*Go posterior +1881.324*Palp troch length -2905.813*Palpal femur length

		+3398.295*Palpal hand width +199.610*Leg I troch length -2312.019*Leg I femur length +1542.288*Leg IV femoropatella length -2482.242*Leg IV tibia length +160.015*Leg IV tibia width +948.361*Leg IV tarsus length
Females (LDA 2)	*L. abditus* sp. nov.	f(x)= -323.386 -1.289*Setae car posterior +1.448*Tergite VIII +7.267*Sternite V +6.182*Go posterior -20.458*Palpal femur length -132.446*Palpal hand width +106.429*Palpal movable finger length -261.425*Leg IV femoropatella width +1450.923*Leg IV tarsus length
	L. chyzeri	f(x)= -382.042 -2.405*Setae car posterior +2.572*Tergite VIII +8.749*Sternite V +7.492*Go posterior -215.529*Palpal femur length +41.933*Palpal hand width -102.619*Palpal movable finger length -462.751*Leg IV femoropatella width +2059.029*Leg IV tarsus length
	L. nodosus	f(x)= -391.437 +2.437*Setae car posterior +6.138*Tergite VIII +4.410*Sternite V +3.650*Go posterior -1023.669*Palpal femur length +671.077*Palpal hand width +91.866*Palpal movable finger length +323.444*Leg IV femoropatella width +2110.035*Leg IV tarsus length
