# Supplementary figures and images for: Integrative Taxonomy Approach Reveals Cryptic Diversity within the Phoretic Pseudoscorpion Genus Lamprochernes (Pseudoscorpiones: Chernetidae)
Source: Insects. 2023 Jan 25;14(2):122. doi: 10.3390/insects14020122 (PMC9964657; doi:10.3390/insects14020122)

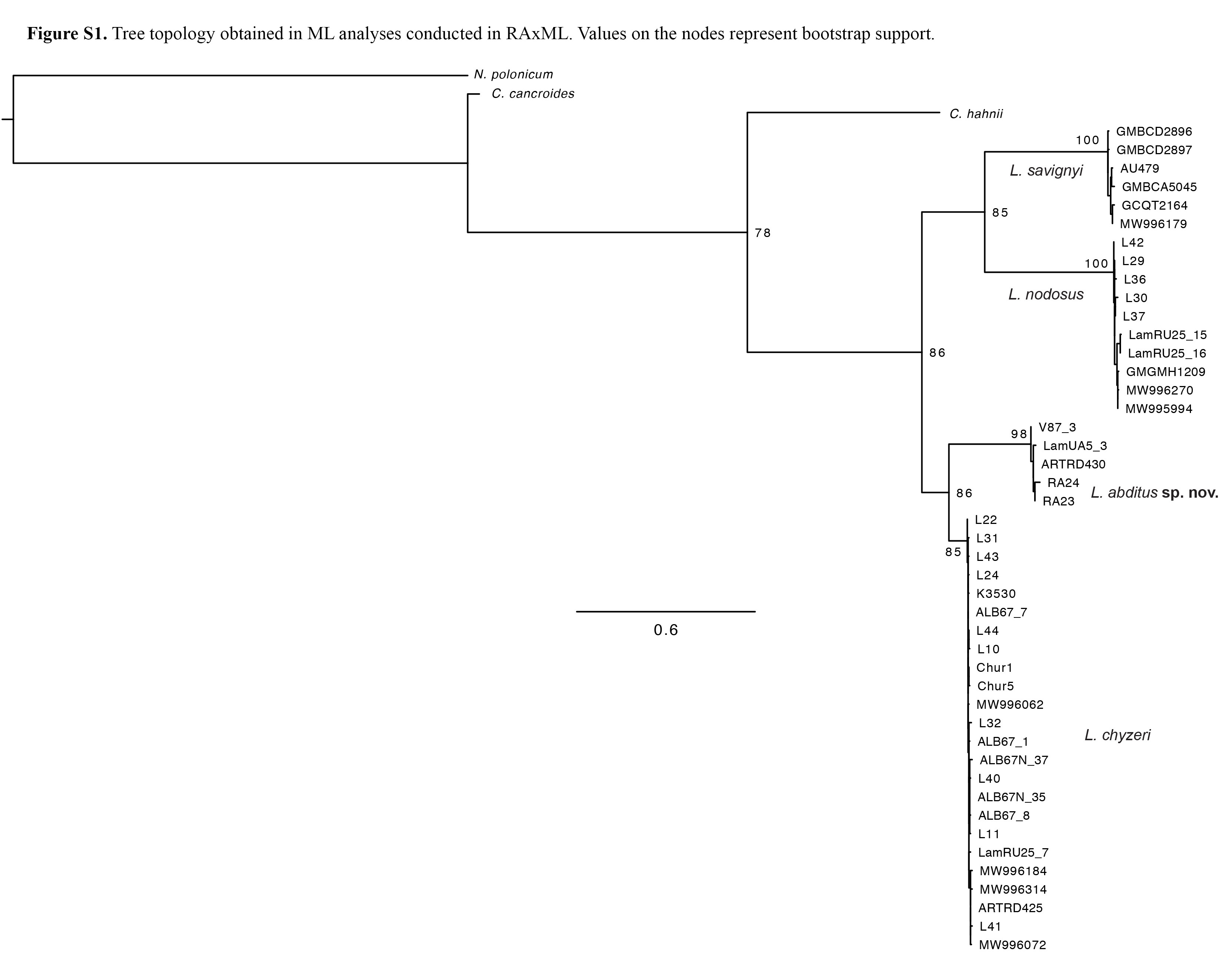

Supplement: Supplementary file 1 [file insects-14-00122-s001.zip › supplementary figures/Figure S1.jpg]

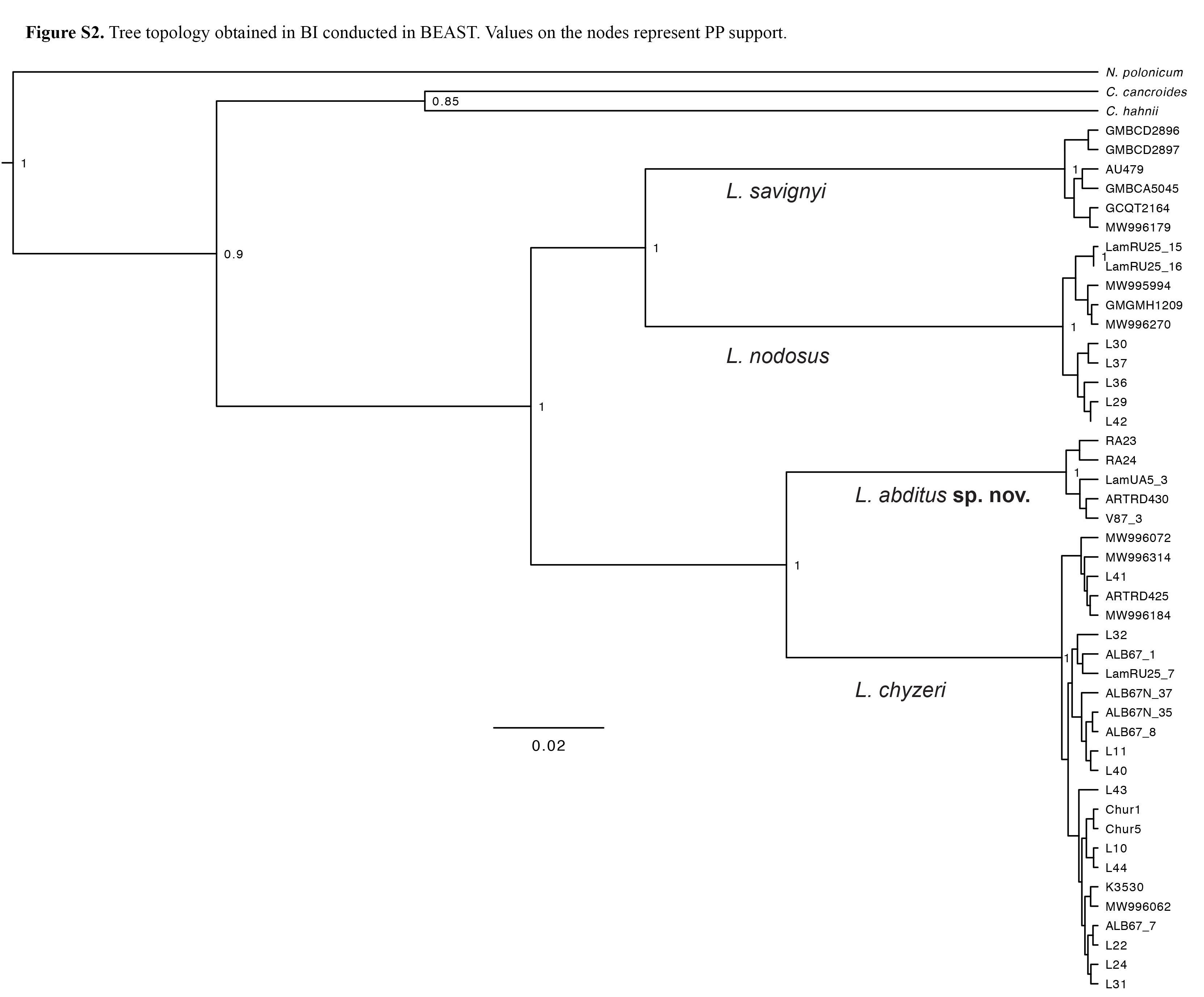

Supplement: Supplementary file 1 [file insects-14-00122-s001.zip › supplementary figures/Figure S2.jpg]

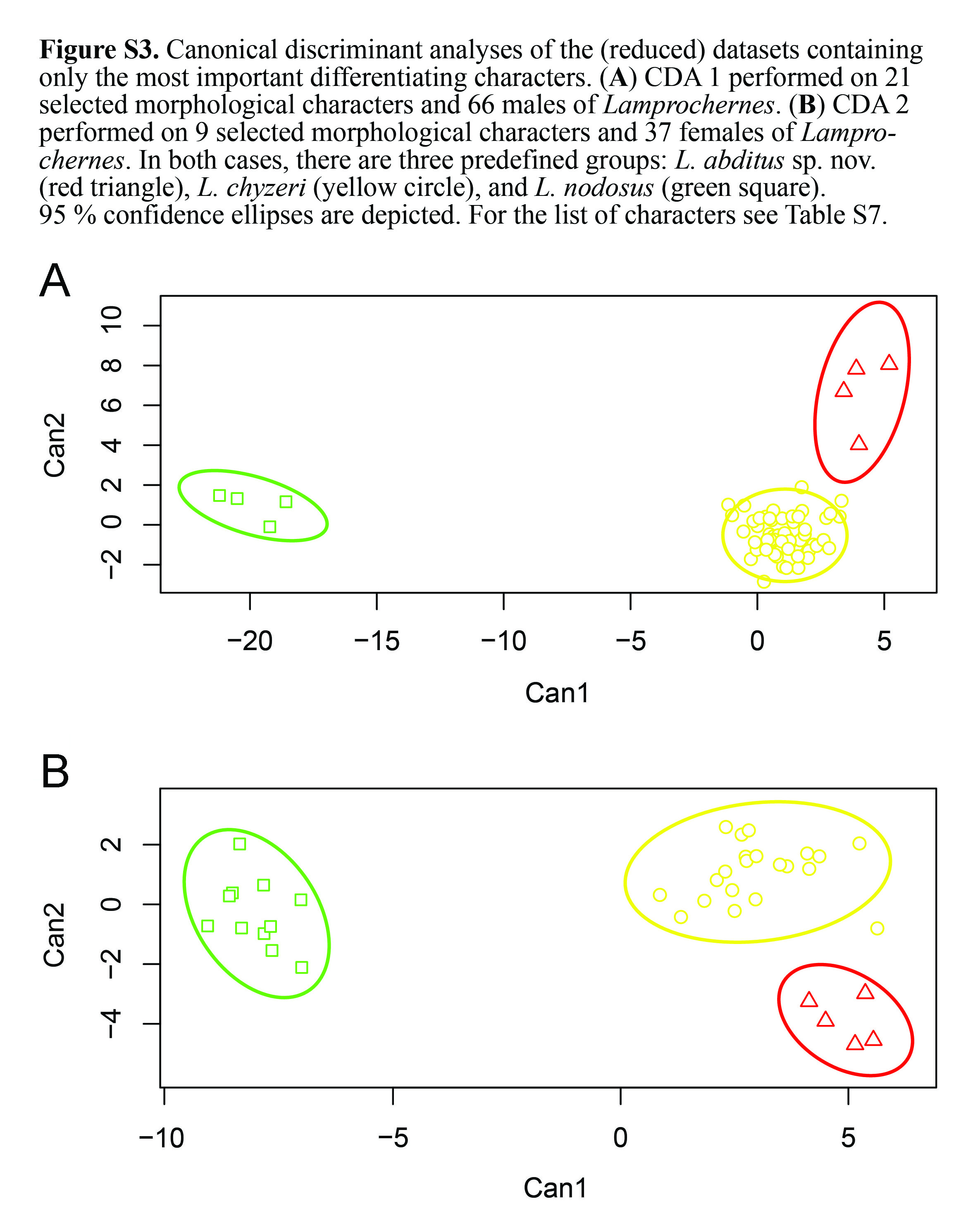

Supplement: Supplementary file 1 [file insects-14-00122-s001.zip › supplementary figures/Figure S3.jpg]
